# Decrease post-transplant relapse using donor-derived expanded NK-cells

**DOI:** 10.1038/s41375-021-01349-4

**Published:** 2021-07-26

**Authors:** Stefan O. Ciurea, Piyanuch Kongtim, Doris Soebbing, Prashant Trikha, Gregory Behbehani, Gabriela Rondon, Amanda Olson, Qaiser Bashir, Alison M. Gulbis, Kaur Indreshpal, Katayoun Rezvani, Elizabeth J. Shpall, Roland Bassett, Kai Cao, Andrew St Martin, Steven Devine, Mary Horowitz, Marcelo Pasquini, Dean A. Lee, Richard E. Champlin

**Affiliations:** 1 Department of Stem Cell Transplantation and Cellular Therapy, The University of Texas MD Anderson Cancer Center, Houston, Texas, USA.; 2 Division of Hematology/Oncology, Department of Medicine, University of California Irvine, Irvine, CA, USA.; 3 Abigail Wexner Research Institute at Nationwide Children’s Hospital, Columbus, OH, USA.; 4 Division of Hematology, The Ohio State University, Columbus, OH, USA.; 5 Division of Pharmacy, The University of Texas MD Anderson Cancer Center, Houston, TX, USA.; 6 GMP Laboratory, The University of Texas MD Anderson Cancer Center, Houston, TX, USA.; 7 Department of Biostatistics, The University of Texas MD Anderson Cancer Center, Houston, TX, USA.; 8 Department of Pathology, The University of Texas MD Anderson Cancer Center, Houston, TX, USA.; 9 Center for International Bone Marrow Transplant Research, Milwaukee, WI, USA.; 10 National Marrow Donor Program, Minneapolis, MN, USA.; 11 These authors contributed equally: Dean A. Lee, Richard E. Champlin.

## Abstract

In this phase I/II clinical trial, we investigated the safety and efficacy of high doses of mb-IL21 ex vivo expanded donor-derived NK cells to decrease relapse in 25 patients with myeloid malignancies receiving haploidentical stem-cell transplantation (HSCT). Three doses of donor NK cells (1 × 10^5^−1 × 10^8^ cells/kg/dose) were administered on days −2, +7, and +28. Results were compared with an independent contemporaneously treated case-matched cohort of 160 patients from the CIBMTR database.

After a median follow-up of 24 months, the 2-year relapse rate was 4% vs. 38% (*p* = 0.014), and disease-free survival (DFS) was 66% vs. 44% (*p* = 0.1) in the cases and controls, respectively. Only one relapse occurred in the study group, in a patient with the high level of donor-specific anti-HLA antibodies (DSA) presented before transplantation. The 2-year relapse and DFS in patients without DSA was 0% vs. 40% and 72% vs. 44%, respectively with HR for DFS in controls of 2.64 (*p* = 0.029). NK cells in recipient blood were increased at day +30 in a dose-dependent manner compared with historical controls, and had a proliferating, mature, highly cytotoxic, NKG2C+/KIR+ phenotype.

Administration of donor-derived expanded NK cells after haploidentical transplantation was safe, associated with NK cell-dominant immune reconstitution early post-transplant, preserved T-cell reconstitution, and improved relapse and DFS. TRIAL REGISTRATION: NCT01904136 (https://clinicaltrials.gov/ct2/show/NCT01904136).

## INTRODUCTION

Allogeneic hematopoietic stem-cell transplantation (HSCT) is the preferred curative treatment strategy for patients with advanced hematologic malignancies [1, 2]. While treatment-related mortality has progressively decreased over time, relapse has remained relatively unchanged since the beginning of transplantation [3, 4], and is now the most common cause of treatment failure. The relapse rate for patients with high-risk acute myeloid leukemia (AML) in remission can be 50% or more based on disease risk and status at transplant [5, 6].

Natural killer (NK) cells are lymphocytes capable of recognizing and eliminating malignant and virally infected cells [7]. Multiple studies have administered NK cells for AML immunotherapy [8]; however, the therapeutic potential has been limited, at least in part because of the relatively low number of NK cells obtained by apheresis [9].

Our group hypothesized that high doses of NK cells obtained by ex vivo expansion could overcome this limitation, and developed an expansion method using K562 feeder cells expressing membrane-bound IL21 and 4–1BBL (FC21) [10]. We previously demonstrated in a phase I clinical trial that three doses of ex vivo expanded haploidentical NK cells generated from the transplant donor could be safely infused early post-transplant at doses up to 1 × 10^8^ cells/kg, without toxicity or adverse effects [11].

Here we report final results of a phase II extension study and long-term follow-up of all patients treated on this clinical trial, as well as results of an independent comparison with contemporaneous case-matched controls performed by the Center for International Blood and Marrow Transplant Research (CIBMTR).

## MATERIALS AND METHODS

### Study design and patients

This single arm, phase I/II study was conducted at the University of Texas MD Anderson Cancer Center (MDACC) between 06/2014 and 07/2019. Patients, 18–65 years of age, with myeloid malignancies and <5% bone marrow blasts were enrolled (Supplemental Protocol).

### Transplant procedure

Haploidentical donors donated 1 unit (500 ml) of blood collected on day −16 for NK-cell production and underwent a bone marrow harvest (goal 3 × 10^8^ TNC/kg), collected and infused on day 0 of transplant.

Donors were selected according to the consensus recommendations [12]. Killer cell immunoglobulin-like receptor (KIR) genotyping and alloreactivity were evaluated in all donor-recipient pairs [11]. NK-cell alloreactivity in the graft-versus-host direction and/or KIR B genotype was preferred for donor selection, but not mandatory [13, 14]. A donor without donor-specific anti-HLA antibodies (DSA) was preferred; however, if no such donor was available, desensitization treatment was performed, as previously described [15, 16].

All patients received conditioning with fludarabine 160 mg/m^2^, melphalan 100–140 mg/m^2^ and 2 Gray of total body irradiation, and post-transplant cyclophosphamide (PTCy)-based GVHD prophylaxis as previously described [11].

The trial was approved by the Institutional Review Board of MDACC and conducted under an Investigational New Drug application from the US Food and Drug Administration (ClinicalTrials.gov number NCT01904136). Patients and donors provided written informed consent according to the Declaration of Helsinki.

### Ex vivo expansion and infusion of NK cells

NK cell expansions were initiated from CD3-depleted blood mononuclear cells and stimulated weekly with FC21 for 14 days under current good manufacturing practice conditions, as previously described [10]. The FC21-NK-cell product was infused fresh on day −2 and thawed from cryopreserved aliquots on days +7 and +28 post transplant. In the previously reported phase I trial, 13 patients were treated with NK cells in escalating doses from 10^5^ cells/kg/dose to 10^8^ cells/kg/dose without dose-limiting toxicities [11], establishing the target dose for the phase II part of the study at 10^8^ NK cells/kg/dose.

### Case-matched analysis with CIBMTR controls

All patients with at least 1-year follow-up were independently matched by a CIBMTR statistician with controls who received first haploidentical transplant with PTCy during the same period. Matching (up to 1:4) was based on age, disease, and disease status at transplant. Analysis was performed for all patients and separately for myeloablative (MAC) and reduced-intensity conditioning (RIC) controls.

### Outcome definitions and statistical analysis

The primary outcome was disease-free survival (DFS). Secondary outcomes included overall survival (OS), cumulative incidence of neutrophil and platelet engraftment, acute GVHD (aGVHD), chronic GVHD (cGVHD), relapse and non-relapse mortality (NRM). All outcomes were computed from the date of stem cell infusion until occurrence of the first outcome event.

The impact of NK-cell therapy on clinical outcomes in comparison with the CIBMTR matched controls was computed using marginal Cox regression models. Patient-, disease- and transplant characteristics that were not included in the matching process were considered for adjustment in the comparison analyses.

### Assessment of immune reconstitution

Clinical flow cytometry was performed from recipient blood samples to assess the number of T-, B-, and NK-cell subsets at 30, 90, 180, and 365 days post transplant. Blood samples for high-parameter mass cytometry were obtained from patients at 7, 14, 21, and 28 days post transplant, and before and after the third dose of NK cells, processed as previously described [11].

Immunophenotyping by mass cytometry with stochastic clustering was performed to compare immune reconstitution in patients to healthy controls and FC21-NK cell products using a 34-parameter panel of heavy-metal conjugated antibodies as summarized in Supplementary Information Table S1.

### Role of the funding source

The funders of the study had no role in study design, data collection, data analysis, data interpretation, or writing of the report. All authors had full access to all the data in the study and had final responsibility for the decision to submit for publication.

## RESULTS

### Patients and transplant characteristics

Twenty-five patients received ex vivo expanded NK cells. Due to a short follow-up of 3 months, 1 patient was excluded from the analyses. This patient was alive and in remission without GVHD at last follow-up. Demographics of the remaining 24 cases are summarized in Table 1. The median age was 46 years (range 18–60 years), 41% of the AML/MDS patients had detectable measurable residual disease at transplant, and 54% had high-risk cytogenetics. The disease risk index was high/very high in 15 patients (63%). Five patients (21%) had high DSA levels (>5000 MFI) before transplant. The median follow-up time for surviving patients was 24 months (range 12–51 months). Clinical outcomes are summarized in Table 2. Donor and recipient KIR characteristics are summarized in Supplementary Information Tables S2–S3.

### NK-cell manufacturing

Characteristics of the NK-cell products of the first 13 patients treated in phase I study were previously described [11]. For 12 patients treated in phase II extension study, the NK cell target dose was achieved in all but one patient (who received 3 × 10^7^ NK cells/kg/dose due to high body weight) (Supplementary Information Table S4). The median expansion was 2995-fold. Only two donor products failed to achieve at least 1000-fold expansion; one was cryopreserved prior to starting the expansion culture, which resulted in poor recovery and viability. The fresh NK cell products had a median viability of 96% (IQR 94.5–97) and purity 99.0% (IQR 97.5–99.8). Feeder cell contamination was <1%, CD19+ cells were detectable in only four products, and T-cell contamination was below 0.1%. The viability (median 89%, IQR 83–93%) and recovery (91%, IQR 87–99%) were excellent in the cryopreserved NK-cell products after thawing for infusion (Supplementary Information Fig. S1).

### Engraftment, graft-versus-host disease, and non-relapse mortality

All patients achieved primary engraftment. Neutrophil engraftment at day 28 and platelet engraftment at day 100 were 100 and 75% (95% CI 55–91), respectively. Day 30 chimerism was evaluated in 23 patients; 21 patients (91%) had full donor and two patients (9%) had mixed chimerism. Four of five patients with DSA received desensitization treatment prior to transplant [16]. Although engrafted, the only patient that relapsed was the patient with DSA who was not desensitized prior to transplant. A higher failure rate was observed in the five patients with DSA, of which one patient relapsed and two died of NRM.

aGVHD occurred in ten patients (nine had grade 2 and one with grade 3/4). The patient who developed severe aGVHD was a male with an older female donor, previously reported by us to be associated with significantly higher incidence of aGVHD in haploidentical transplants [17]. No patients developed cGVHD.

### Relapse and survival post transplant

**Fig. 1:**
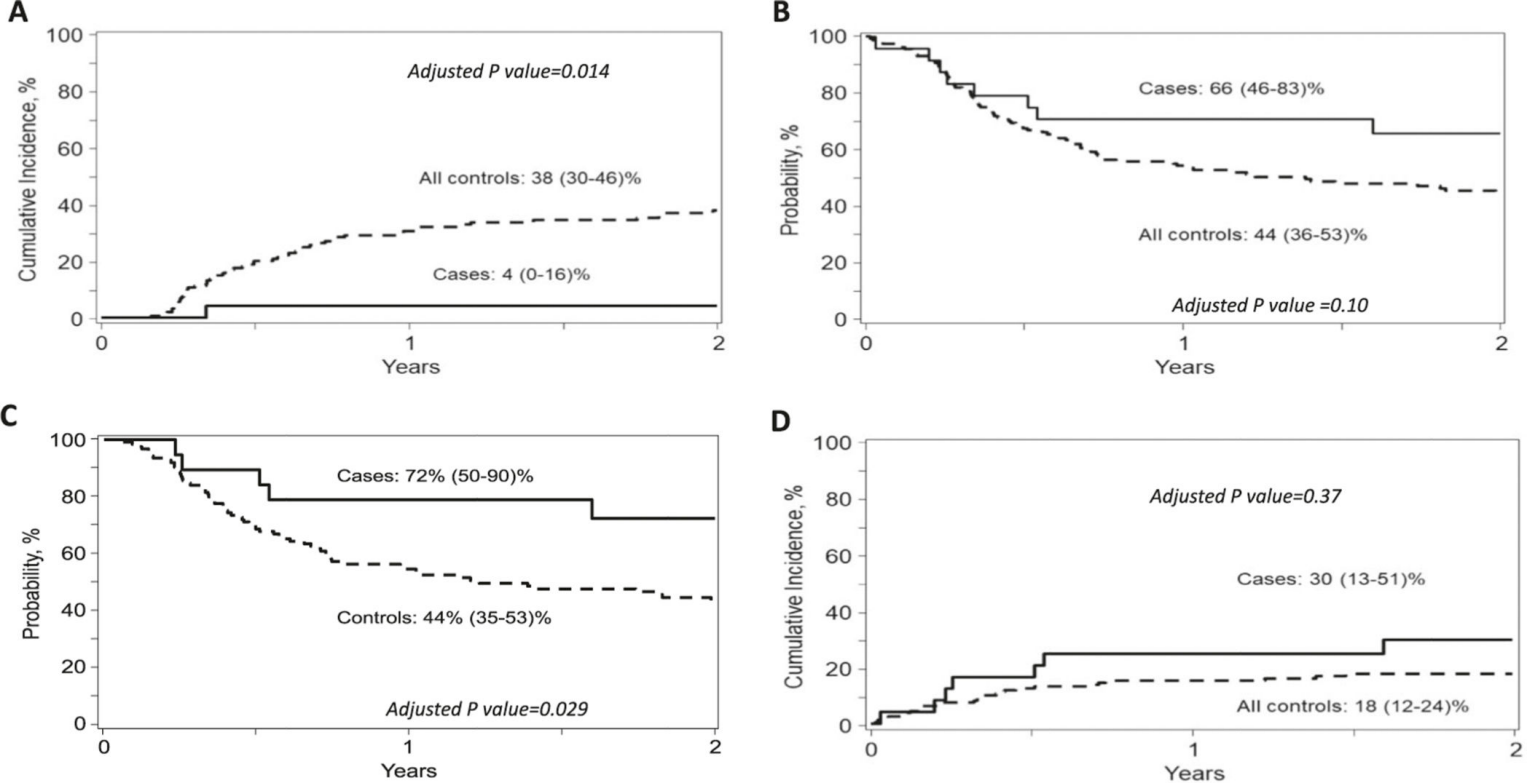
Transplant outcomes of NK cell treatment group (cases) and the CIBMTR control group. **A** Relapse between cases and controls with the cumulative incidence at 2 years of 4% (95% CI 0–16) vs. 38% (95% CI 30–46), respectively (adjusted *P* value = 0.014). **B** DFS of cases vs. controls with the probability at 2 years of 66% (95% CI 51–87) vs. 44% (95% CI 36–53), respectively (adjusted *P* value = 0.10). **C** DFS of cases and controls who did not have DSA before transplant. A significantly higher DFS was observed in patients without DSA treated with NK cell infusion compared with controls without DSA with probability at 2 years of 72% vs. 44%, respectively (adjusted *P* = 0.029). D NRM of cases vs. controls with the probability at 2 years of 30% (95% CI 13–51) vs. 18% (95% CI 12–24), respectively (adjusted *P* value = 0.37).

Only 1 patient relapsed, resulting in a very low cumulative incidence of relapse (4% at 2 years; 95% CI 0–16) (Fig. 1A). The 1-and 2-year probability of DFS were 71% (95% CI 51–87) and 66% (95% CI 51–87), respectively (Fig. 1B), whereas 1- and 2-year OS were 75% (95% CI 56–90) and 70% (95% CI 50–86), respectively. Among 19 patients without DSA, the 2-year probability of both DFS and OS was 72% (95% CI 50–90) (Fig. 1C). NRM for all treated patients at 100 days, 1 year and 2 years were 16.7% (95% CI 5.2–33.7%), 25% (95% CI 10–44), and 30% (95% CI 13–51), respectively (Fig. 1D), and 21% (95% CI 6–42) and 28% (95% CI 9–51), respectively for patients without DSA.

Causes of death are described in Supplementary Information Table S5. None of the patients treated on study died from disease relapse as compared with 36 of 67 patients (54%) of those who died in the control group.

### Immunologic reconstitution and viral reactivation

Lymphocyte subsets were evaluated from blood samples collected at days 30, 90, 180, and 360 post transplant (Supplementary Information Table S6). The mean absolute NK-cell count at day 30 (before the third NK-cell infusion) was 636 cells/mm^3^ (standard deviation; SD 964). Patients who received the highest NK-cell dose (10^8^/kg/dose) had a mean absolute NK-cell number of 1084 (SD 1282) cells/mm^3^, compared to 284 (SD 305) cells/mm^3^ for those receiving 1 × 10^7^−3 × 10^7^/Kg/dose, and 122 (SD 136) cells/mm^3^ for <1 × 10^7^/Kg/dose (*p* = 0.064).

**Fig. 2:**
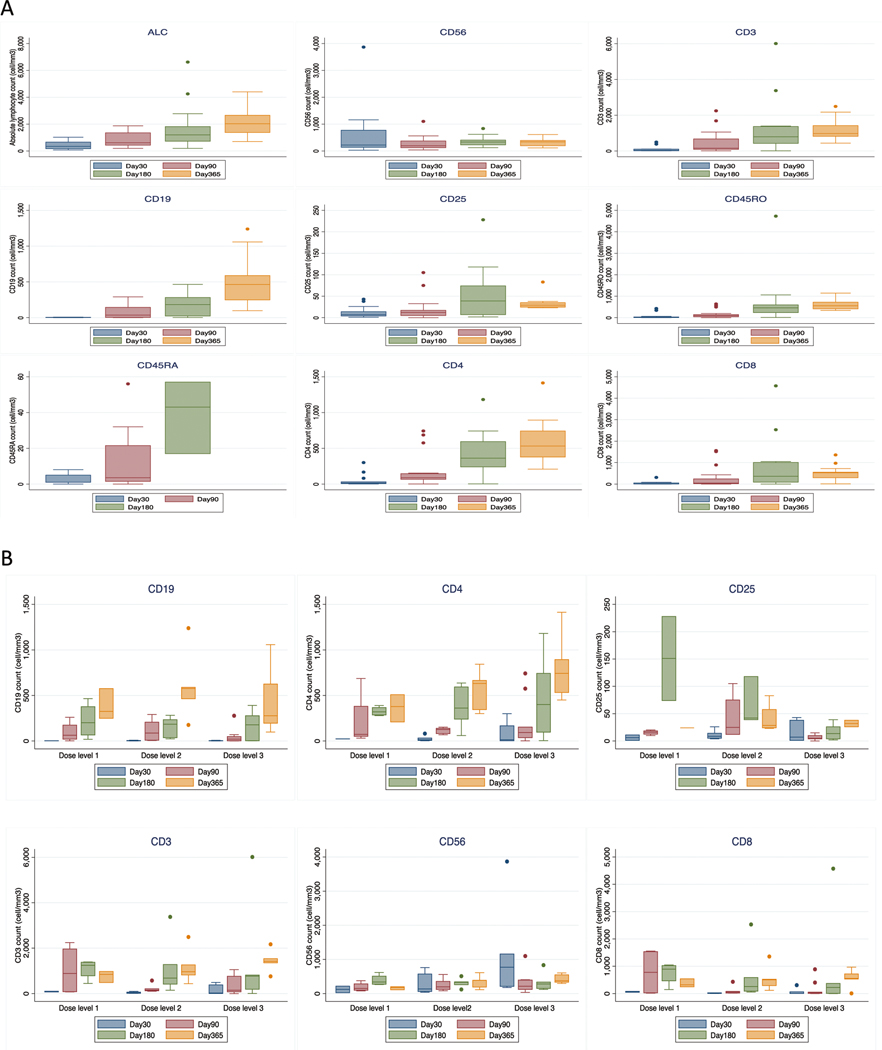
Immunologic reconstitution of patients treated on the clinical trial. **A** The median number of absolute lymphocytes (ALC), CD56+, CD3+, CD4+, CD8+, CD19+, CD25+, CD45RO, and CD45RA cells at 30, 90, 180, and 360 days after transplant. Number of all lymphocyte subsets gradually increased and returned to normal by day 90 after transplant. **B** Number of B and T cell subsets after transplant stratified by NK cell dose level. High number of CD56+ cells was observed in patients who received high NK cell dose (dose level 3: 1 × 10^8^/Kg/dose) compared with low (dose level 1: <1 × 10^7^/Kg/dose) and intermediate dose (dose level 2: 1 × 10^7^−3 × 10^7^/Kg/dose) (*P*= 0.064). At day 90 and 180, a significantly lower number of CD25+ cells in patients who received higher NK cell dose was observed when compared with low and intermediate dose. The mean number of CD25+ cells at day 90 for patients who received low, intermediate, and high NK dose was 15 cells/mm^3^ (SD 4.2), 42 cells/mm^3^ (SD 38) and 6.3 cells/mm^3^ (SD 5.1), respectively (*P* = 0.005), and at day 180 were 151 cells/mm^3^ (SD 108), 66 cells/mm^3^ (SD 44) and 16 cells/mm^3^ (SD 14), respectively (*P* = 0.025). No significant difference in number of CD19+, CD4+, CD8+, and CD3+ cells between patients who received different dose of NK cells. Bars and whiskers represent median ± interquartile range.

In the phase I study, CD8+ T cell numbers were lower at day 30 compared to controls [11]. Here, we confirmed low CD8+ numbers until day 90. In addition, significantly lower CD25+ cell numbers at day 90 and 180 was observed with higher NK-cell doses than in lower doses. No other significant differences in mean numbers of CD19+, CD4+, CD8+, and CD3+ cells in patients who received different dose levels were observed. At day 180 post transplant, all lymphocyte subsets were fully recovered (Fig. 2).

**Fig. 3:**
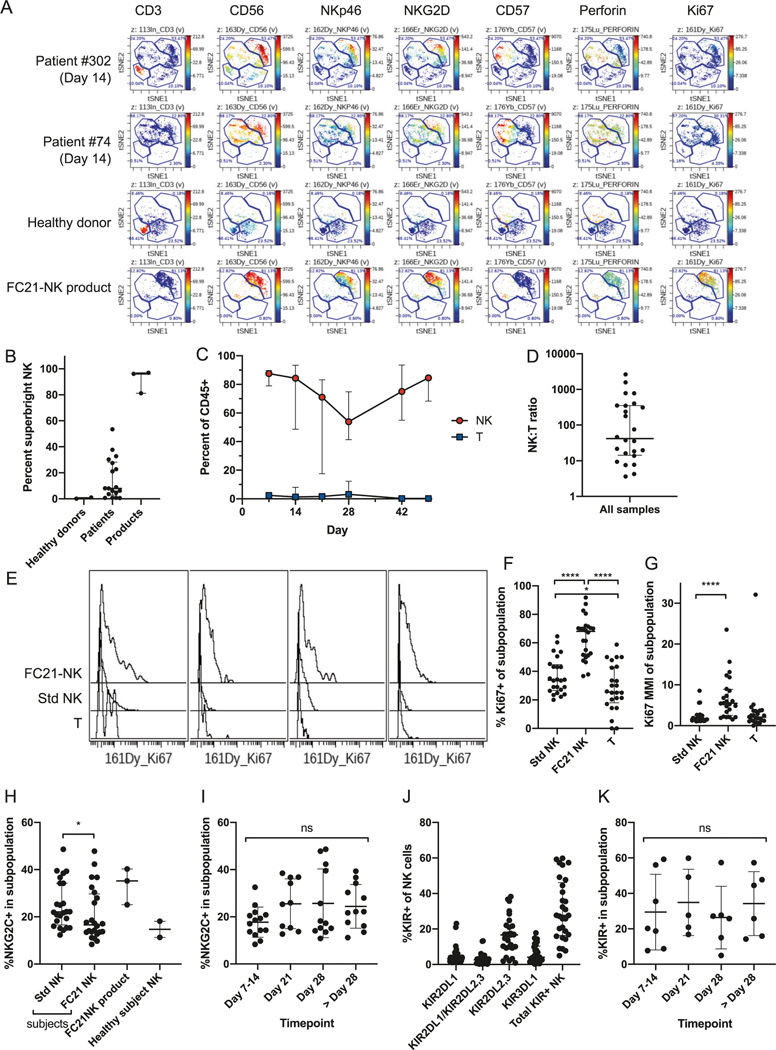
Immunophenotyping by mass cytometry with stochastic clustering to compare immune reconstitution in patients compared to healthy controls and FC21-NK cell products. Blood was obtained at the indicated timepoints, and mononuclear cells (MNC) were isolated, processed, and labeled with a 34-parameter panel of heavy-metal conjugated antibodies (Supplementary Information Table S1), along with healthy subject MNC and expanded FC21-NK cell products as controls. Events were collected on a CyTOF 2 mass cytometer (Fluidigm). Events were filtered by sequential gating on live, singlet (event length vs. 191Ir), non-apoptotic (PARP-negative), and hematopoietic (CD45+) cells, and then clustered by visual interactive stochastic neighbor embedding (ViSNE, CytoBank) on CD3, TIGIT, NKP30, NKP46, CD56, NKG2D, CD94, and CD57, using equal sampling to unbias differences in sample event number. ViSNE clusters corresponding to T-cells, standard NK cells, FC21-NK cells, and any remaining MNC were created using the reference samples. The percentage of cells in clusters 1 through 4 were quantified for each sample. Ki67 staining within the gated populations was determined as a surrogate for proliferation. **A** shows representative plots from two patients, one healthy subject, and one NK cell product, showing expression of key activating surface markers, perforin, and Ki67 across four broad phenotypic clusters identified. Cluster 1 (bottom left) consisting of CD3^+^ T cells, cluster 2 (top middle) of CD3^−^CD56^dim^NKG2D^dim^CD57^+^ “standard” NK cells, cluster 3 (top right) consisting of CD56^br^NKG2D^br^NKp46^br^CD57^−^ (“superbright”) NK cells corresponding to the phenotype of the infused FC21-NK cell product, and cluster 4 (bottom middle) consisting of all remaining cells. Cluster 3 identifies a unique phenotypic signature associated with the FC21-NK cells that is not present in healthy subjects and persists in patients at day 14 (7 days after adoptive transfer) and later (Supplementary Information Fig. S2). **B** NK cell immune reconstitution in patients over time maintains FC21-NK “superbright” phenotype with high proliferation, expressed as percent of total cell events, of cluster 3 (superbright FC21-NK cells) in healthy donors, FC21-NK cell products, and in patients receiving FC21-NK cell products (across all timepoints). **C** Proportion of Cluster 1 (T cells) and total NK cells (Cluster 2 + Cluster 3) in blood of study subjects across time. **D** The ratio of NK cells and T cells for all patients and timepoints assessed (*n* = 24). **E** Ki67 staining in FC21-NK cells (Cluster 3), standard NK cells (Cluster 4), and T cells (Cluster 1) in four representative patient samples obtained at day 14 (7 days after the NK cell infusion at day 7). **F, G** The percent of Ki67+ and Ki67 mean metal intensity (MMI), respectively, in standard NK cells, FC21-NK cells, and T cells for all patients and timepoints assessed (*n* = 24). Bars and whiskers represent median ± interquartile range, *P* < 0.0001. **H** NK cells in clusters 2 (standard NK) and 3 (FC21-NK) as assessed for expression of NKG2C across all patients at all timepoints, with FC21-NK cell infusion products and healthy subjects shown for reference. **I** the percent of NKG2C + NK cells from all patients at all timepoints, with early (Days 7 and 14) and late (>day 28) timepoints pooled. **J** NK cells (CD3−/CD56+) gated and assessed for KIR expression and summed for total percentage of KIR + NK cells, and then **K** plotted across time, with early and late timepoints pooled.

We also assessed quantitative and phenotypic NK-cell reconstitution after transplant using mass cytometry and compared with FC21-NK-cell infusion products and healthy subjects as reference (Fig. 3, Supplementary Information Fig. S2). Using this approach to define the variegated receptor repertoires of NK cells, we identified two distinct phenotypic clusters that contained the majority of the NK-cell populations presented in healthy donor blood and FC21-NK-cell products, respectively (Fig. 3A). The FC21-NK cells were “superbright” compared to normal NK-cells in expression of CD56, NKp46, and NKG2D, were negative for CD57, contained moderate levels of perforin, and were highly proliferative as assessed by Ki67 expression. We then used these clusters to quantify relative proportions of these cells in patients after FC21-NK-cell adoptive transfer and identified up to 50% of NK cells in blood of patients expressing this “superbright” phenotype (Fig. 3B, C). The NK:T-cell ratio was >1 for all patient samples at all timepoints (Fig. 3D). Moreover, the “superbright” cluster 3 NK cells appeared more proliferative than T-cells or standard phenotype NK cells (Fig. 3E), by both percent of cells that were Ki67+ (Fig. 3F) and overall Ki67 expression (mean metal intensity, MMI, Fig. 3G).

Since FC21-NK cells are known to have high expression of NKG2C and KIR [10, 18], which are hallmarks of mature licensing and are minimally expressed by endogenous NK cells that arise during post-transplant reconstitution [19, 20], we evaluated NKG2C and KIR expression in our patients. Both the standard and FC21-NK-cell clusters contained a large percentage of NKG2C+ cells (Fig. 3H). In contrast to previous reports in haploidentical transplants with PTCy [18, 19], we observed that NKG2C+ NK cells (median 18.89%, IQR 14.28–31.96%, Fig. 3I) and KIR + NK cells (median 27.63%, IQR 16.38–46.10%, Fig. 3J) were consistently high across all timepoints throughout the post-transplant period, with no significant differences between the timepoints assessed. Similarly, we observed high levels of expression of all inhibitory KIRs in NK cells with the expected dominance of KIR2DL2/KIR2DL3 (Fig. 3K).

In addition to immune recovery, we evaluated viral reactivation post-transplant. Only 5/23 patients (22%) had BK virus cystitis (all grade 1), and only 9/23 (39%) had CMV reactivation (>137 copies/ml).

### Comparison with CIBMTR controls

A total of 160 matched controls were selected (81 and 79 patients that received MAC and RIC regimens, respectively). Similar to our cases (21%), 35 patients (22%) had DSA before transplant. Baseline characteristics were comparable between cases and controls except more patients in the treatment group had adverse cytogenetic risk (54% vs. 28%) (Table 1). Detailed comparative outcomes are summarized in Table 2. The 2-year probabilities of relapse, NRM, DFS, OS for all CIBMTR controls were 38% (95% CI 30–40), 18% (95% CI 12–24), 44% (95% CI 36–53), 58% (95% CI 49–66), respectively.

Results from multivariable analysis showed that NK-cell administration was associated with a significantly decreased risk of disease relapse vs. controls (HR of controls 13.76; 95% CI 1.70–111.24, *p* = 0.014) (Fig. 1A). Other outcomes comparing cases and controls are presented in Table 2. The benefit of NK cell administration in reducing relapse and improved survival was more significant when compared with RIC controls (HR of controls 14.18; 95% CI 1.75–115.0, *p* = 0.013 for relapse and 2.28; 95% CI 1.08–4.82, *p* = 0.03 for DFS) (Supplementary Information Table S7), whereas there was a trend towards improved relapse (HR of controls 7.24; 95% CI 0.95–55.16, *p* = 0.06) and DFS (HR of controls 1.55; 95% CI 0.67–3.55, *p* = 0.30) when compared with MAC controls (Supplementary Information Table S8). However, when patients with DSA were excluded from analysis (uniformly excluded in HSCT trials), the 2-year cumulative incidence of relapse was 0%, 40% (95% CI 31–49), 29% (95% CI 18–41) and 51% (95% CI 38–64) for cases, controls, MAC controls and RIC controls, respectively, whereas DFS was 72% (95% CI 50–90), 44% (95% CI 35–53), 50% (95% CI 37–62) and 37% (95% CI 25–51), respectively. A better DFS was noted in the study group without DSA compared with all controls without DSA in multivariable analysis with adjusted HR of controls of 2.64 (95% CI 1.10–6.33; *p* = 0.029) (Fig. 1C). Results from multivariable analyses for MAC and RIC controls, with and without DSA, are described in the Supplementary Information Tables S7–S10.

## DISCUSSION

Here we report results of a phase I/II clinical trial and long-term follow-up for patients receiving multiple high doses of NK cells administered early post-transplant to decrease relapse and improve survival. Administration of NK cells was very well tolerated with no increased toxicities or adverse effects including GVHD, which make this cell type ideal for use after transplant. Only one patient developed severe GVHD, an older female donor for a male recipient. Older female donors were associated with much higher incidence of severe aGVHD (50% vs. 10% for other donors), as recently reported by our group [17].

NK cells were manufactured from the same haploidentical transplant donor, which could have potential advantages as there is no risk of rejecting the cells by the graft. Infusion of higher NK-cell doses was associated with progressively higher NK-cell numbers early post-transplant, suggesting a dose-dependent effect. In addition, a shift in early immune reconstitution of effector cell subsets to highly functional NK cells was noted.

Using this approach, the relapse rate was remarkably low (4%) for treatment group vs. 30 and 46% for patients receiving MAC and RIC conditioning in the CIBMTR database. In our trial, only one patient relapsed, a patient with high DSA levels against donor HLA antigens who did not receive desensitization treatment. Pre-existing DSA may have rejected donor NK cells, which might have decreased the anti-tumor effect in this patient [15].

Previous studies have shown a very low total number of NK cells as well as NKG2C+ subsets early post-haploidentical transplant with PTCy [19, 21], and significant reconstitution of NKG2C+ NK cells did not occur until 6 months post transplant [19]. These studies also showed that KIR+ NK cells arising from in vivo expansion of mature NK cells in the graft are depleted by PTCy, and single-KIR-expressing NK cells become almost undetectable by day 30 post transplant, as immature endogenous KIR^neg^ NK cells arise. Patients with KIR+ NK cells above the median at day 30 had a significantly higher risk of relapse [19]. In our cohort, we observed ~100-fold higher NK-cell numbers by day 30, which correlated with NK-cell dose, and were associated with a functional improvement and dramatic decrease in relapse rate post transplant.

The mechanisms and predictive factors for NK cell control of relapse remain unclear. Several patients had KIR-ligand-mismatched and/or KIR B donors, but a majority did not. With only one relapse observed, this suggests that NK-cell alloreactivity may not be required in this setting. Our group has shown that these highly activated and cytotoxic “superbright” NK cells have a unique phenotype with upregulated CD56 expression (CD56^bright^) and increased CD16 (atypical of traditional blood NK cells), increased cell surface activating receptors (NKp30, NKp44, DNAM-1, NKG2D, and others). They are hyperfunctional compared with NK cells from PB, possessing high cytotoxicity and cytokine production [10, 22].

Despite suppressing early CD8+ T-cell reconstitution, we observed no increase in CMV reactivation and a remarkably low incidence of BK virus hemorrhagic cystitis. None of the patients experienced grade 2–4 hemorrhagic cystitis as compared with ~40% historically using the same conditioning regimen [23].

In conclusion, we have shown that high doses of donor-derived FC21-NK cells can be safely administered after haploidentical transplant, are associated with an NK cell-dominant immune reconstitution in the early post-transplant period, and a very low relapse in patients with myeloid malignancies.

## Supplementary Material

1739240_Sup_Material_1

1739240_Sup_Info

## Figures and Tables

**Table 1.** Demographics and clinical characteristics of the patients and CIBMTR controls.

	Cases *N* (%)	Controls *N* (%)	MAC controls *N* (%)	RIC controls *N* (%)
Number of patients	24	160	81	79
Number of centers	1	61	38	40
Age at transplant, years				
Median (range)	46 (18–60)	44 (19–60)	45 (19–60)	43 (19–61)
18–30	4 (17)	37 (23)	20 (24)	17 (22)
31–40	3 (13)	26 (16)	12 (15)	14 (18)
41–50	8 (33)	31 (19)	15 (19)	16 (20)
51–60	9 (38)	66 (41)	34 (42)	32 (41)
Gender				
Male	12 (50)	84 (53)	39 (48)	45 (57)
Female	12 (50)	76 (48)	42 (52)	34 (43)
Race				
Caucasian	17 (71)	89 (56)	47 (58)	42 (53)
African American	4 (17)	48 (30)	23 (28)	25 (32)
Asian	2 (8)	13 (8)	6 (7)	7 (9)
Other	1 (4)	1 (1)	0	1 (1)
Missing	0	9 (6)	5 (6)	4 (5)
Ethnicity				
Hispanic or Latino	4 (17)	25 (16)	14 (17)	11 (14)
Non-Hispanic or non-Latino	20 (83)	132 (83)	65 (80)	67 (85)
Missing	0	3 (2)	2 (2)	1 (1)
Performance score				
90–100	18 (75)	78 (49)	34 (42)	44 (56)
<90	6 (25)	82 (51)	47 (58)	35 (44)
HCT-CI				
0	5 (21)	30 (19)	12 (15)	18 (23)
1	3 (13)	29 (18)	14 (17)	15 (19)
2	4 (17)	31 (19)	15 (19)	16 (20)
3	6 (25)	31 (19)	21 (26)	10 (13)
>3	6 (25)	39 (24)	19 (23)	20 (25)
CMV serostatus				
Negative	2 (8)	41 (26)	18 (22)	23 (29)
Positive	22 (92)	119 (74)	63 (78)	56 (71)
Donor-specific antibodies	5 (21)	35 (22)	18 (22)	17 (22)
Disease				
AML	13 (54)	104 (65)	52 (64)	52 (66)
CML	7 (29)	24 (15)	13 (16)	11 (14)
MDS	4 (17)	32 (20)	16 (20)	16 (20)
Cytogenetic risk for AML—number of patients^a^	13	136	68	68
Favorable	0	15 (11)	8 (12)	7 (10)
Intermediate	6 (46)	77 (57)	35 (51)	42 (62)
Adverse	7 (54)	38 (28)	22 (32)	16 (24)
Not reported	0	6 (4)	3 (4)	3 (4)
Disease status at transplant—AML				
First complete remission	10 (77)	80 (77)	40 (77)	40 (77)
Relapse	3 (23)	24 (23)	12 (23)	12 (23)
Disease status at transplant—CML				
First chronic phase	4 (57)	13 (54)	4 (31)	9 (82)
Second chronic phase	3 (43)	11 (46)	9 (69)	2 (18)
Disease status at transplant—MDS				
Advanced stage	4 (100)	32 (100)	16 (100)	16 (100)
Graft type				
Bone marrow	24 (100)	42 (26)	15 (19)	27 (34)
Blood	0	118 (74)	66 (81)	52 (66)
Conditioning intensity				
MAC	0	81 (51)	81	0
RIC/NMA	24 (100)	79 (49)	0	79
MAC regimens				
TBI + Cy + Flud	0	1 (1)	1 (1)	0
TBI + Flud	0	39 (48)	39 (48)	0
TBI + Cy	0	2 (2)	2 (2)	0
Bu + Cy + Flud	0	17 (21)	17 (21)	0
Bu + Cy	0	4 (5)	4 (5)	0
Bu + Flud + Mel	0	2 (2)	2 (2)	0
Bu + Flud	0	11 (14)	11 (14)	0
Cy + Flud + Mel	0	2 (2)	2 (2)	0
Cy + Flud	0	2 (2)	2 (2)	0
TBI + other	0	1 (1)	1 (1)	0
RIC/NMA regimens				
TBI + Cy + Flud	0	64 (81)	0	64 (81)
TBI + Flud + Mel	21 (88)	3 (4)	0	3 (4)
Flud + Mel	3 (13)	3 (4)	0	3 (4)
TBI + Bu + Flud	0	5 (6)	0	5 (6)
Bu + Flud	0	2 (3)	0	2 (3)
Cy + Flud	0	2 (3)	0	2 (3)
GVHD prophylaxis				
PTCy + TAC + MMF	24 (100)	160 (100)	81 (100)	79 (100)
Year of transplant				
2014	2 (8)	27 (17)	14 (17)	13 (16)
2015	9 (38)	49 (31)	25 (31)	24 (30)
2016	5 (21)	33 (21)	13 (16)	20 (25)
2017	4 (17)	33 (21)	19 (23)	14 (18)
2018	4 (17)	18 (11)	10 (12)	8 (10)
Median follow-up of survivors (range), months	24 (12–51)	36 (3–59)	35 (6–59)	36 (3–49)

*MAC* myeloablative conditioning, *RIC* reduced intensity conditioning, *HCT-CI* hematopoietic cell transplant comorbidity index, *CMV* cytomegalovirus, *AML* acute myelogenous leukemia, *MDS* myelodysplastic syndrome, *TBI* total body irradiation, *Cy* cyclophosphamide, *Flud* fludarabine, *Bu* busulfan, *Mel* melphalan, *PTCy* post-transplant cyclophosphamide, *TAC* tacrolimus, *MMF* mycophenolate mofetil.

a Cytogenetic risk for AML was defined as follows:

Favorable: t(15:17), inv(16), del(16q), t(16:16), t(8:21) without del(9q) or complex.

Intermediate: normal karyotype, +6, +8, −Y, del(12p), 11q23, t(9:11).

Adverse/poor: complex karyotype, −5/del(5q), −7/del(7q), abnormal (3q, 9q, 11q, 21q, 17p), t(6:9), t(9:22).

**Table 2.** Post-transplant outcomes and multivariable analysis of cases and controls with and without DSA.

All cases and controls	Cases (*N* = 24) (95% CI)	Controls (*N* = 160) (95% CI)	MAC controls (*N* = 81) (95% CI)	RIC controls (*N* = 79) (95% CI)
Neutrophil recovery				
28 days	100%	89 (83–93)%	98 (74–100)%	84 (74–91)%
Platelet recovery				
100 days	75 (55–91)%	88 (82–93)%	91 (84–97)%	84 (74–92)%
Total acute GVHD^a^	10 (41.7)	57 (35.6)	27 (33.3)	30 (38)
Grade 1	0	9 (5.6)	3 (3.7)	6 (7.6)
Grade 2	9 (37.5)	24 (15)	12 (14.8)	12 (15.2)
Grade 3	0	20 (12.5)	10 (12.3)	10 (12.7)
Grade 4	1 (4.2)	4 (2.5)	2 (2.5)	2 (2.5)
Chronic GVHD				
1 year	0%	39 (31–47)%	39 (28–50)%	39 (28–51)%
2 years	0%	44 (35–52)%	45 (34–57)%	41 (30–54)%
Chronic GVHD severity				
Limited	0%	13 (8)	2 (2)	11 (14)
Extensive	0%	51 (32)	34 (42)	17 (22)
DFS				
1 year	71 (51–87)%	54 (46–62)%	60 (49–70)%	48 (37–60)%
2 years	66 (46–83)%	44 (36–53)%	49 (38–60)%	40 (28–51)%
OS				
1 year	75 (56–90)%	69 (61–76)%	69 (58–79)%	69 (58–79)%
2 years	70 (50–86)%	58 (49–66)%	61 (50–72)%	54 (42–66)%
Relapse				
1 year	4 (0–16)%	31 (24–38)%	24 (15–34)%	37 (27–49)%
2 years	4 (0–16)%	38 (30–46)%	30 (20–41)%	46 (34–58)%
NRM				
1 year	25 (10–44)%	15 (10–21)%	16 (9–25)%	14 (7–23)%
2 years	30 (13–51)%	18 (12–24)%	21 (12–31)%	14 (7–23)%
Cases and controls without DSA	Cases (*N* = 19) (95% CI)	Controls (*N* = 125) (95% CI)	MAC controls (*N* = 63) (95% CI)	RIC controls (*N*=62) (95% CI)
DFS				
1 year	79 (58–94)%	54 (45–63)%	61 (48–73)%	48 (35–60)%
2 years	72 (50–90)%	44 (35–53)%	50 (37–62)%	37 (25–51)%
OS				
1 year	79 (58–94)%	71 (63–79)%	70 (57–81)%	73 (61–83)%
2 years	72 (50–90)%	60 (51–69)%	64 (51–76)%	56 (43–69)%
Relapse				
1 year	0%	31 (23–40)%	21 (12–33)%	41 (28–54)%
Cases and controls without DSA	Cases (*N* = 19) (95% CI)	Controls *N* = 125)	MAC (95% CI) controls (*N* = 63) (95% CI)	RIC controls (*N* = 62) (95% CI)
2 years	0%	40 (31–49)%	29 (18–41)%	51 (38–64)%
NRM				
1 year	21 (6–42)%	15 (9–21)%	18 (9–28)%	12 (5–21)%
2 years	28 (9–51)%	17 (10–24)%	21 (12–33)%	12 (5–21)%
Multivariate analysis^b,c^				
All cases and controls	Number events/Evaluable	Hazard ratio (95% confidence interval)		*P* value
DFS				
Cases	8/24	1.00^d^		
Controls	87/160	1.89 (0.89–4.02)		0.10
NRM				
Cases	7/24	1.00^d^		
Controls	28/160	0.68 (0.28–1.60)		0.37
Relapse^e^				
Cases	1/24	1.00^d^		
Controls	59/160	13.76 (1.70–111.24)		0.014
OS				
Cases	7/24	1.00^d^		
Controls	67/160	1.48 (0.66 – 3.30)		0.34
Cases and controls without DSA^f^		Number events/Evaluable	Hazard ratio (95% confidence interval)	*P* value
DFS				
Cases		5/19	1.00^d^	
Controls		69/125	2.64 (1.10–6.33)	0.029
NRM				
Cases		5/19	1.00^d^	
Controls		21/125	0.77 (0.32–1.89)	0.57
OS				
Cases		5/19	1.00^d^	
Controls		51/125	1.63 (0.66–4.01)	0.29

*DSA* donor-specific anti-HLA antibodies, *MAC* myeloablative conditioning, *RIC* reduced intensity conditioning, *GVHD* graft-versus-host disease, *DFS* disease-free survival, *OS* overall survival, *NRM* non-relapse mortality.

a Sample size is too small to provide a cumulative incidence of acute GVHD, therefore numbers and percentages are presented.

b Variables considered for analysis: recipient age, recipient gender, recipient race and ethnicity, HCT-CI, KPS, CMV serostatus, graft type, conditioning intensity, and year of transplant.

c Conditioning intensity was a significant factor in the Relapse model. The results shown for DFS, NRM and OS are from the models with only the main effect.

d Reference group.

e Adjusted for conditioning intensity.

f Since there was 0 relapse event in the cases without DSA, we were unable to perform multivariable analysis for relapse.
