# Supplementary material for: Decrease post-transplant relapse using donor-derived expanded NK-cells
Source: Leukemia. Author manuscript; Available in PMC 2022 Oct 7. (PMC8727305; doi:10.1038/s41375-021-01349-4)
Supplement: 1739240_Sup_Material_1 [file NIHMS1739240-supplement-1739240_Sup_Material_1.pdf]

PDOL Working Copy

THE UNIVERSITY OF TEXAS
MD Anderson Cancer Center

Protocol Page

A Phase I/II Clinical Trial of NK cells Administration to Prevent Disease Relapse for Patient with High-risk Myeloid Malignancies Undergoing Haploidentical Stem-cell Transplantation
2012-0708

Core Protocol Information

**Short Title**	NK Cells to Prevent Disease Relapse for Patients High Risk Myeloid Malignancies
**Study Chair:**	Stefan Ciurea
**Additional Contact:**	Doris R. Soebbing<br>Peggy S. LeCompte
**Department:**	Stem Cell Transplantation and Cellular Therapy
**Phone:**	713-792-8750
**Unit:**	423
**Study Manager:**	Doris R. Soebbing
**Full Title:**	A Phase I/II Clinical Trial of NK cells Administration to Prevent Disease Relapse for Patient with High-risk Myeloid Malignancies Undergoing Haploidentical Stem-cell Transplantation
**Public Description:**	The goal of this clinical research study is to learn about the safety of giving a kind of immune cells called "natural killer" (NK) cells with standard chemotherapy and an allogeneic stem cell transplant (using cells from a donor). Researchers also want to learn the highest tolerable dose of NK cells that can be given.<br>Immune system cells (such as NK cells) are made by the body to attack foreign or cancerous cells. Researchers believe that the NK cells you receive from the donor may react against the leukemia cells in your body, help eliminate leukemia, and prevent disease relapse.
**Protocol Type:**	Standard Protocol
**Protocol Phase:**	Phase I/Phase II
**Version Status:**	Working copy
**Version:**	36

Which Committee will review this protocol?

◉ The Clinical Research Committee - (CRC)

Protocol Body

1.0 Objectives

1. **Primary objective:**
    1. To evaluate safety, tolerability and identify the maximum tolerated dose (MTD) of expanded NK cells to be used in patients with myeloid malignancies undergoing a haploidentical stem-cell transplant.

2. **Secondary objectives:**
    1. To determine survival of NK cells *in vivo* post-transplant.
    2. To determine the function of NK cells post-transplant and compare with a retrospective control treated with no NK cells.
    3. To estimate the proportion of patients with engraftment/graft failure.
    4. To estimate the non-relapse mortality (NRM) at day 100 post transplant.
    5. To estimate the cumulative incidence of grade III-IV aGVHD at day 100.
    6. To assess the rate of chronic GVHD within the first year post transplantation.
    7. To assess immune reconstitution post transplant.
    8. To assess disease response, disease-free survival (DFS) and overall survival (OS) after transplantation.
    9. To perform a retrospective comparison of patients treated on the study with NK cells will be performed with a Center for International Blood and Bone Marrow Research (CIBMTR) control of similar patients who did not receive NK cells.

2.0 Background

Haploidentical stem-cell transplantation:

Haploidentical stem cell transplantation (HaploSCT) is an attractive form of transplantation for patients lacking an HLA identical sibling donor, due to the immediate availability, ease of stem cell procurement and the possibility to further collect donor cells for cellular therapy. Historically, HaploSCT has been limited by the high rates of graft rejection and acute graft-versus-host disease (aGVHD) elicited by the presence of donor T-cells in the haploidentical graft, while a strong anti-tumor effect was observed.[(1)]

We have previously shown that, in T-cell depleted (TCD) haploidentical stem cell transplantation, the conditioning regimen consisting of fludarabine, melphalan and thiotepa (FMT) was very effective in clearing the bone marrow of leukemia, inducing remission in the great majority of patients with relapse or refractory disease. More than 90% of AML/MDS patients achieved remission after transplant in this study; however, almost all patients with advanced disease at the time of transplant relapsed. This could be attributed, at least in part, to a poor graft-versus-tumor effect related to the lack of T-cells in the graft. [(2)]

Recently, we have changed our approach to haploidentical transplantation by using a T-cell replete (TCR) graft and high-dose post transplant cyclophosphamide (Cy) for

GVHD prevention, followed by immunosuppression with tacrolimus and mycophenolate. Administration of high-dose cyclophosphamide early post transplantation in combination with tacrolimus and mycophenolate mofetil has proven safe, and produced engraftment and GVHD rates similar to HLA-matched sibling transplants.[3, 4]

We are investigating the use of post-transplant Cy in a phase II clinical trial ongoing at MD Anderson Cancer Center. To date, more than 40 patients were treated, and outcomes for the first 32 consecutive patients were initially reported in abstract format. [5] TCR HaploSCT patients received the same conditioning regimen (FMT) as previously reported by us in TCD HaploSCT and we compared the outcomes between these 2 groups of patients.

After a median follow-up of approximately 12 months for the TCR group (and 44 months for the TCD group), the outcomes of the patients treated with TCR haploidentical transplants were significantly better, due to better immune reconstitution in the first 6 months post transplant.[5]

Primary engraftment was achieved in 94% in the TCR group and 81% in the TCD group (p=0.1). Day-100 non-relapse mortality (NRM) was 12% in the TCR group vs. 21% in the TCD group, and at 1 year was 16% vs. 42% (p=0.02), respectively. Progression-free survival (PFS) was 50% vs. 21% (p=0.02). For patients treated while in remission from their malignancy, treatment-related mortality was 0% vs. 67% (p=0.001) and PFS 82% vs. 25% (p=0.01). The cumulative incidence of grade II-IV aGVHD was 20% vs. 11% (p=0.2) and cGVHD 7% vs. 18% (p=0.03). Actuarial OS and PFS at 1 year post-transplant were 64% and 30% (p=0.02) and 50% vs. 21% (p=0.02) for the TCR vs. TCD groups, respectively. For patients in remission, survival was 92% vs. 33%, p=0.02) and PFS 82% vs. 25% (p=0.01).[5]

Improved NRM in the TCR group was related to significantly better immunologic reconstitution of T-cell subsets. On day 30 post transplant, there was a significantly better recovery of absolute CD4 cells in the TCR group (median 26 vs. 1, p=0.00084) and CD8 cells (median 22 vs. 1, p=0.006). CD4 cells remained significantly lower in the TCD group until after day 180 when the median CD4 count was 194 vs. 69 in the TCR group (p=0.04), while the difference in CD8 counts became non-significantly higher in the TCR after day 90 (median 167 vs. 54, p=0.4).[5] Similar results are seen when analysis was restricted for pates with AML only between the two groups (analysis in progress).

These results suggested that, using the same conditioning regimen, TCR HaploSCT is a safer approach with lower treatment-related mortality and improved outcomes compared with TCD HaploSCT. We have decided to use this approach in future studies of haploidentical transplants. While outcomes have improved primarily based on improving treatment-related mortality, disease relapse post transplant remains a concern, especially for patients with high risk disease or not in remission at transplant. This provides on opportunity to use NK cells in an attempt to decrease relapse rate after haploidentical transplants.

Figure 1. Cumulative incidence of NRM for all pts (left) and OS and PFS for pts in remission at transplant (right) treated with TCD and TCR HaploSCT.

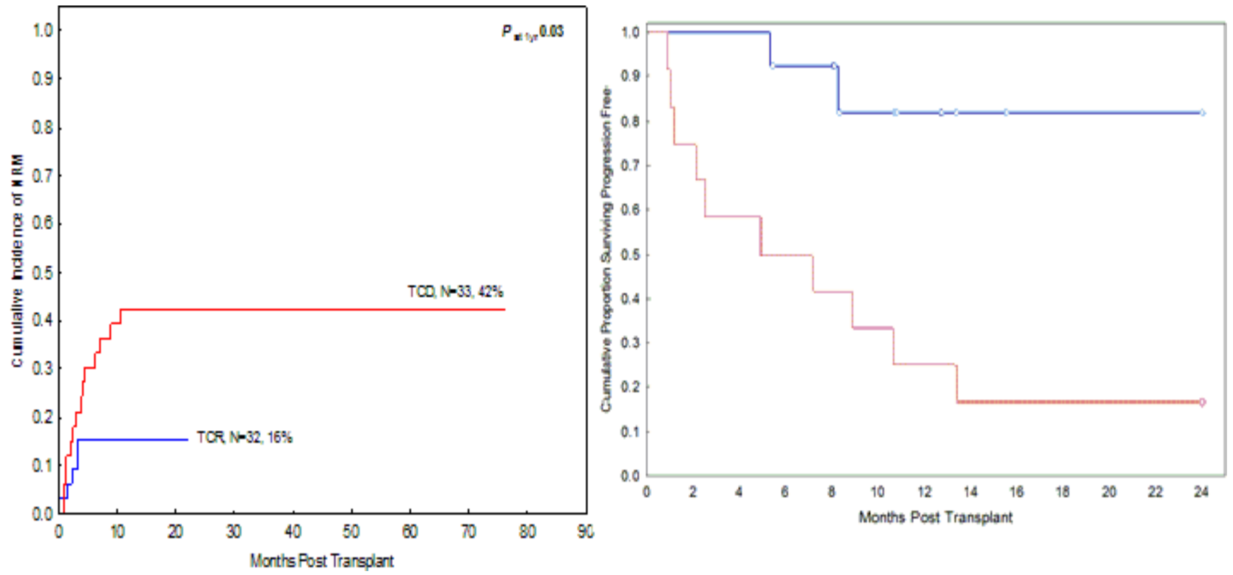

Natural Killer Cells

Natural killer (NK) cells are a unique class of lymphocytes, with cytotoxic, and immunoregulatory function which can mediate potent antileukemia effects.[(6-8)] NK cells are regulated by KIR receptor-ligand interactions and mediate cytotoxicity against certain HLA class I mismatched targets.[(9)] Alloreactive HLA haploidentical NK cells have been reported to enhance engraftment, reduce GVHD and prevent relapse of leukemia post transplant. [(6-8)] This study will utilize NK cells from the same donors as stem cells as adjuvant treatment to eradicate leukemia and prevent disease relapse after transplantation.

Natural killer cells have both cytotoxic and immunoregulatory functions. Human NK cells are characterized by expression of CD56 with or without CD16, and the absence of T-cell receptor and CD3 expression.[(7, 8)] There are 2 subtypes of NK cells: CD56dim CD16bright cells, which compose 90% of the NK cells from circulation which have cytotoxic function, and express MHC class I allele-specific Killer-cell Immunoglobulin-like Receptors (KIR), and the CD56brightCD16neg cells present in small numbers in the peripheral blood but 100% in lymphoid tissues, and secrete immunoregulatory cytokines, like interferon gamma, and promote adaptive immune responses. NK cell cytotoxicity can be upregulated by increased secretion of cytokines including interleukin (IL)-2 (IL-2) and IL-15, whereas IL-12 and IL-18 increase INF-ã secretion by NK cells.[(8)]

NK cell reactivity is governed by activating and inhibitory receptors. By the “missing ligand model” initially described by Karre in 1986, killing of tumor cells by NK cells

occurs when there is a missing signal (or missing self) on the target cells.[10] Several inhibitory receptors and putative activation receptors of the NK have been characterized and they can be classified as lectin-like receptors (CD94) receptors and KIR.[11-13] In man, some HLA class I molecules expressed on the target cells are ligands of the CD94 and KIR receptors. The interactions between NK receptors molecules are specific for different HLA isotypes, for example, the inhibitory receptor KIR2 DL4 interacts with HLA-G molecules. KIRs interact with groups of alleles of HLA-C and HLA-B. It is of interest that the KIR2DL1 receptor interacts with alleles of the HLA-C locus that have the amino acid lysine (Lys) at residue 80 (C-lys-80) while the receptors KIR2DL2 and KIR2DL3 interact with alleles of HLA-C that have asparagine (Asn) at residue 80 (C-asn-80). Interestingly, the substitutions Lys/Asn at residue 80 define a bi-allelic system in all alleles of HLA-C in which all the alleles of this locus carry either of these amino acids. The alleles of HLA-B locus present also different capacities to interact with the receptor KIR3DL1; only the HLA-B alleles carrying the supertypic serologic epitope HLA-Bw4 (encoded in the amino acid stretch spanning residues 77-83) interact with KIR3DL1.[11-13] Therefore, in an allogeneic transplantation setting, in which the donor and recipient are mismatched in the alleles of HLA-B or in HLA-C, the NK cells from the donor may not be inhibited by the target cells of the recipient that lack the appropriate HLA alleles which interact with the KIR receptors. In allogeneic bone marrow transplants the graft-versus-leukemia effect has been attributed in part to NK cells not inhibited because of the lack of the appropriate HLA ligand in the recipient.

It is important to note that the KIR genes are encoded as a haplotype block (KIR loci) on chromosome 19q. The KIR haplotypes differ in gene content between individuals and some individuals may lack some KIR genes. The NK repertoire therefore may vary from individual to individual and will depend on the HLA phenotype and in the KIR haplotype gene composition. It is generally accepted, that in order to avoid auto-reactivity, the NK repertoire is developed when both, the inhibitory receptor and its specific HLA ligand are present and expressed in the same individual.

In the proposed study, we will use NK cells from the same haploidentical stem-cell donor. The genotype KIR analysis of the donors paired with the analysis of HLA alleles of the donor and the mismatched alleles (absent) of the patient will allow the evaluation of the potential NK anti-leukemia repertoire for each donor-recipient pair (Appendix C).

Allogeneic stem cell transplantation confers an immune mediated graft-vs.-leukemia effect. NK cells have been shown to mediate a potent antileukemia effect against myeloid leukemias. As indicated above, KIRs recognize HLA-C and -Bw antigens, when the corresponding ligand (HLA molecule) is present, cytotoxicity is inhibited; when the ligand is absent, cytotoxicity occurs (based on the “missing ligand” principle). Thus, NK cytotoxicity is most marked when NK cells react against haploidentical target missing the relevant class of HLA class I molecules. Velardi et al. have studied the results of T-cell depleted haploidentical transplants from alloreactive or nonalloreactive donors based upon this “missing ligand” principle, and demonstrated that patients predicted to have alloreactive NK cells had no relapses compared to a 75% relapse in recipients of nonalloreactive transplants.[14] The positive effect of KIR ligand incompatibility has been

best seen with haploidentical transplants.

Clinical experience using infusion of NK cells after transplant:

Several lines of evidence suggest that increased numbers of alloreactive NK cells would have benefit in the transplant setting, despite the fact that retrospective results vary depending on patient population, underlying diseases, conditioning regimens, graft composition and cell dose, degree of T-cell depletion, post-transplant immunosuppressive regimens and differing methods of determining alloreactivity. Ruggeri et al. proposed the infusion of NK cells after transplant to target disease relapse based on a retrospective evaluation of NK alloreactivity in transplanted patients [(14)] and supportive NK models. This group analyzed 92 patients with high risk leukemia who received a haplotype mismatched hematopoietic stem cell transplant (HSCT) and reported that NK cell alloreactivity improved engraftment, protected from graft-versus-host disease (GVHD) and reduced the rate of relapse. In mouse xenograft models it was demonstrated the role of alloreactive NK cells in eradicating human leukemia, improved engraftment by targeting host T lymphocytes, and reduced GVHD by eliminating recipient-type dendritic cells.[(15)] In addition, the NK cell dose of the infusion product has been associated with better outcomes following matched sibling transplants.[(16)]

A number of small prospective studies published mostly in abstract format (listed in Table 1), have reported on the safety of NK cell infusion. This experience has been recently reviewed by us.[(8)] While no dose-limiting toxicities have been described, none of the studies in the post-transplant setting were powered to assess the therapeutic effect of the NK cells. The only study published in manuscript format that reported on a higher number of patients infused with NK cells post transplant, was a study reported by the Duke University group on non-expanded NK cells.[(17)] This group infused a total of 51 NK cell-enriched donor lymphocyte infusions (DLIs) to 30 patients following a 3-6/6 HLA matched T-cell-depleted non-myeloablative allogeneic transplant using with alemtuzumab part of the conditioning regimen.[(17)] Eight weeks following transplantation, donor NK cell-enriched DLIs were processed using a CD56+ selecting column, with up to 3 fresh infusions allowed. Fourteen matched and 16 haploidentical transplant patients received a total of 51 NK cell-enriched DLIs. The median number of CD3- CD56+ NK cells infused was 10.6×10^{6} cells/kg and 9.21×10^{6} cells/kg for matched and mismatched, respectively. The median number of contaminating CD3+CD56- T-cells infused was 0.53×10^{6} and 0.27×10^{6} in the matched and mismatched setting, respectively. All but 2 subjects had donor engraftment accounting for >80% of their hematopoiesis at the time of first infusion. After a median follow-up of 12 months for matched sibling donor transplants, and 27 months for the haploidentical transplants, 1 year overall survival was 43% and 42%, respectively.[(15)] Evaluating outcomes by disease, 1-year survival was 50% for the 19 patients with myeloid diseases 29% for patients with lymphoid diseases. Only 1 patient each in the matched (n=14) or mismatched (n=16) groups experienced severe aGVHD with little other toxicity attributable to the infusions. Importantly, long-term responders had improved T-cell phenotypic recovery and improved duration of responses and overall survival after multiple NK cell-enriched infusions.[(17)]

Table 1. Clinical studies using adoptive NK cell immunotherapy after hematopoietic stem cell transplantation.

First author	Disease and # of treated patients	Type of transplant (n)	Median NK cell dose infused	Method of generation of NK cell product	Phase of clinical trial	aGVHD Y/N	Response/outcome
Koehl	AML (1), ALL (4), HD (1), RMS (1)	Haplo (7)	N/A	Unstimulated leukapheresis, CD3 depletion and CD56 selection, IL2 activation	I	Y (3 pts)	5/7 patients were alive in CR 4-13 months post SCT
Passweg	AML (4), CML (1)	Haplo (5)	N/A	Unstimulated leukapheresis, CD3 depletion and CD56 selection	I	N	3/5 pts were alive and CR 18-36 months after NK infusion
Slavin	MDS (2), AML (1), ALL (1), biphenotypic (1), HD (1); NHL (2).	Haplo (3)<br>MRD (4)<br>MUD (1)	2.1x106/kg	CD56 selection from rIL-2 activated lymphocytes	I	N	One pt with relapsed ALL and one with MDS had CR. 4 pts alive, 3 disease free 9-22 month post SCT.
Barkholt	Solid tumors (4), CLL (1)	MRD (5)	13.2x106/kg	Expanded NK cells and NK cell-like T cells	I	N	CLL patient progressed
Uharek	AML (16), ALL (5), CML (2), HD (1), MDS (1)	Haplo (25)	9.6x106/kg	NK cells isolated from CD34- cell fraction; CD3 depletion and CD56 selection	I/II	Y (4 pts)	9/25 patients alive and in CR, with 2-year OS of 29%
Rizzieri	30 patients with myeloid and lymphoid malignancies	Haplo (16)<br>MRD (14)	10.6106/kg	Unstimulated leukapheresis enriched in NK cells using CD56 antibody	I/II	Y (14 pts, 6 /14 had grade I skin aGVHD)	Total of 16 patients in CR with 1 year OS of 43% and 42% of matched and mismatched respectively

Legend: MRD – matched related donor transplant; MUD – matched unrelated, Haplo – haploidentical transplant, AML – acute myeloid leukemia, ALL – acute lymphoblastic leukemia, HD – Hodgkin's disease, RMS – rhabdomyosarcoma, CML – chronic myeloid leukemia, MDS – myelodysplastic syndrome, NHL – non-Hodgkin's lymphoma, CLL – chronic lymphocytic leukemia; Y – yes, N – no; GVHD – graft-versus-host disease. (Adapted from Farhan S, et al. NK cell therapy: targeting disease relapse after hematopoietic stem cell transplantation. Immunotherapy. 2012;4:305.)

Our group recently reported in abstract format the preliminary results from a phase I study demonstrating the safety of haploidentical alloreactive NK cell infusion after allogeneic matched related and unrelated donor transplants added to the preparative regimen.[16] Patients received escalating doses of NK cells (10^6/kg to $3x10^7$/kg) followed by infusion of IL-2 for 5 days. All patients had rapid engraftment and tolerated the treatment well. Only 3 patients developed grade 2 aGVHD while none had grade 3-4 aGVHD. Only one patient died of treatment-related mortality. This trial confirmed that infusion of haploidentical NK cells post transplant is feasible and was not associated with increased incidence of GVHD, delayed engraftment, or higher treatment-related mortality (at NK cell doses of 10^6/kg to $3x10^7$/kg).[18]

***Ex vivo* expansion of NK cells:**

The major obstacle for adoptive NK cell immunotherapy has been obtaining sufficient cell numbers for cell therapy, as these cells represent a small fraction of peripheral white blood cells, propagate poorly *ex vivo*, and have limited life spans *in vivo* . Thus *ex vivo* expansion of NK cells is likely needed to maximize the therapeutic potential of this approach. Common gamma-chain cytokines are important in NK cell activation, maturation, and proliferation. Miller et al. have described improved ex vivo expansion with soluble cytokines, artificial antigen presenting cells (aAPC), and aAPC engineered with co-stimulatory molecules and/or membrane-bound IL-15 (mIL-15).[(19)] Our group generated a membrane-bound IL-21 fusion protein (mIL-21), and found superior *ex vivo* expansion of NK cells when stimulated with K562 aAPC genetically modified to express mIL21 and the co-stimulatory molecules CD86 and CD137L. Freshly isolated peripheral blood mononuclear cells (PBMC) are co-cultured with irradiated K562 aAPC at a ratio of 2:1 (aAPC:PBMC) in the presence of 50 IU/ml of rhIL-2 and then re-stimulated with aAPC every seven days at ratio of 1:1.[(20)]

K562-mIL-21 aAPCs were able to promote a mean NK-cell expansion of 37,200-fold by day 21, with 85% of donors achieving at least 5,000-fold expansion (Appendix D; Figure 1 - Ex vivo expansion of NK cells). Expanded cells expressed very high CD16 levels, NCR levels, and retained the pre-expansion KIR repertoire (Appendix D; Figure 2 - Phenotype of *ex vivo* expanded NK cells). These cells and showed high cytotoxicity to tumor targets and ADCC participation (Appendix D; Figure 3 - Cytotoxicity of expanded NK cells). Thus, clinically significant NK cell expansion from small peripheral blood samples is possible using aAPCs expressing mIL-21.[(20)]

There is limited experience of infusion of expanded NK cells in patients. Five patients were treated at the NCI (PI Crystal Mackall) with 10^5/kg expanded NK cells using IL-15. Two patients had significant GVHD which responded to steroids. The other 3 patients tolerated the infusions very well with no adverse effects. All patients had solid tumors and received T-cell depleted transplants with no post-transplant immunosuppression. We are planning to use post transplant cyclophosphamide, tacrolimus and mycophenolate for GVHD prophylaxis which will likely eliminate or dampen any GVHD reactions, attributed mostly to infusion of contaminating T cells.

NK cells regenerating post transplant are functionally immature:

NK cells generated post transplant have an immature phenotype with low KIR expression and overexpression of NKG2A. During the first 6-9 months post transplant, NK cells gradually acquire phenotypic characteristics of mature cells.[(21)] The ratio of CD56bright/CD56dim cells was found to be reversed for the first 4-6 months post transplant with increase production of interferon gamma, while the numbers of cytotoxic NK cells were strongly reduced. Moreover, the killing potential of NK cells derived from transplanted patients was 4 fold reduced compared with the those of NK cells isolated from normal donor.[(22)]

Nguyen and colleagues analyzed recovery of function of NK cells in patients treated

with T-cell depleted haploidentical transplants and found important phenotypic and functional abnormalities in the first 3-6 months post transplant.[23] During the first 3 months, CD56brightCD3- population was a small fraction of NK cells in donors, while this fraction was 10 times higher in recipients. The recipients' NK cells had lower KIR expression and increased expression of CD94/NKG2A while the NK killing potential of K526 cell line was much lower than in donor counterparts. This group concluded that NK cells generated post T-cell depleted haploidentical transplant had an immature phenotype and poor effector against leukemia cell lines in the first 6 months post-transplant. Strikingly, 7/10 relapses occurred in the immediate post transplant period, all relapses occurred in the first 6 months post-transplant, and median time to relapse was 4 months.[23]

Wang and colleagues evaluated the function of NK cells differentiated and cultured from healthy donors in the presence of immunosuppressive medication (cyclosporin). Cyclosporin treated cultures showed fewer CD56+CD16+KIR+ NK cells while there was a reciprocal increase in CD56+CD16-KIR- NK cells, and increase in INFã producing cells. However, NK cells cultured in the presence of cyclosporin retained cytotoxicity against K562, Raji and KIR ligand expressing lymphoblastoid cells, suggesting the effector stage of cytotoxicity was not affected.[24]

Several retrospective clinical studies evaluated the risk of disease relapse post-transplant in relationship with recovery of NK cells post-transplant and the presence of KIR ligand mismatch between the donor and recipient.[25, 26] A high NK cell count on day 30 or 60 post transplant has been associated with improved outcomes.[25, 26]

These studies suggested that, early post-transplant, NK cells are functionally deficient and patients who had higher NK cell numbers in the early post-transplant period had improved outcomes.

Rationale for the current study:

Disease relapse represents the most important cause of treatment failure after hematopoietic stem-cell transplantation for patients with AML/MDS. Patients with high-risk disease and those that do not achieve remission prior to transplant have higher risk of relapse after transplant. Preventing disease relapse post transplant represents the most important therapeutic approach, and NK cell therapy is one of the most promising interventions to do that.

Preclinical studies have suggested that NK cells generated early post-transplant are functionally deficient. With this new study, we propose to infuse *ex vivo* expanded NK cells, and aim to compensate for decreased in NK cell function early post-transplant. Our hypothesis is that infusion of mature, fully functional NK cells generated *ex vivo* will enhance the antitumor effect of the graft and decrease the rate of relapse post-transplant. We will identify the dose of NK cells suitable for clinical use in haploidentical transplantation in patients with high-risk myeloid malignancies including

AML, MDS, and blast-phase CML. A decrease in relapse rate will be appreciated by comparing outcomes of this group with a retrospective control of patients treated with the same conditioning regimen and no NK cells on the current protocol 2009-0266. If successful, this may set the stage for future use of NK cells after hematopoietic stem cell transplantation.

3.0 Patient Eligibility

3.1 Inclusion criteria

1. Patients age 18 to 65 years old. Eligibility for pediatric patients will be determined in conjunction with an MDACC pediatrician. Patients age 2-17 years old may be enrolled after at least 4 adults (ages 18-65 years old) have been treated without toxicity, as defined in the Statistical Considerations section.

2. Patient with no matched related donor who has a related haploidentical donor identified (</= 7/8 allele match at the A, B, C,DR loci) or transplant is urgently indicated who is willing to undergo a bone marrow harvest and an NK cell collection approximately 2 weeks of the recipient's admission for transplant. The donor must be 16 years of age or older and weigh at least 110 pounds.

3. Patients with one of the following diseases:

    3.1 Acute myeloid leukemia (AML):

    a. First complete remission with high-risk features defined as:
    (i) Greater than 1 cycle of induction therapy required to achieve remission;
    (ii) Preceding myelodysplastic syndrome (MDS);
    (iii) Presence of FLT3 mutations or internal tandem duplication or other mutations associated with poor-risk AML (e.g. DNMT3A, TET2); (iv) FAB M6 or M7 classification; (v) Adverse cytogenetics: -5, del 5q, -7, del7q, abnormalities involving 3q, 9q, 11q, 20q, 21q, 17, +8 or complex karyotype [> 3 abnormalities]; ELN2017 intermediate or high-risk;
    (vi) Treatment-related AML~~,~~;
    (vii) Patients with detectable minimal residual disease in the bone marrow biopsy prior to transplant, or

    b. Second or greater remission; patients beyond second remission have to be in CR at transplant to be eligible, or

    c. Primary induction failure with partial response to therapy who achieve adequate cytoreduction.

    3.2 Patients with myelodysplastic syndromes (MDS):

    a. De novo MDS with intermediate or high-risk IPSS scores. Patients with intermediate-1 features should have failed to respond to hypomethylating agent therapy, or

    b. Patients with treatment-related MDS.

    3.3 Chronic myeloid leukemia (CML):

a. Failed to achieve cytogenetic remission or have cytogenetic relapse after treatment with at least 2 tyrosine kinase inhibitors, or
b. Accelerated phase or blast phase at any time.

4. Performance score of at least 70% by Karnofsky or 0 to 1 by ECOG (age $\geq$ 12 years), or Lansky Play-Performance Scale of at least 70% or greater (age <12 years).

5. Adequate major organ system function as demonstrated by:
   a. Serum creatinine clearance equal or more than 50 ml/min (calculated with Cockroft-Gault formula).
   b. Bilirubin equal or less than 1.5 mg/dl except for Gilbert's disease. ALT or AST equal or less than 200 IU/ml for adults. Conjugated (direct) bilirubin less than 2x upper limit of normal.
   c. Left ventricular ejection fraction equal or greater than 40%.
   d. Diffusing capacity for carbon monoxide (DLCO) equal or greater than 50% predicted corrected for hemoglobin. For children $\leq$ 7 years of age who are unable to perform PFT, oxygen saturation $\geq$ 92% on room air by pulse oximetry.

6. Patient or patient's legal representative, parent(s) or guardian should provide written informed consent. Assent of a minor if participant's age is at least seven and less than eighteen years.

3.2 Exclusion criteria

1. HIV positive; active hepatitis B or C.
2. Uncontrolled infections; PI is the final arbiter of this criterion.
3. Liver cirrhosis.
4. CNS involvement within 3 months.
5. Positive pregnancy test in a woman with child bearing potential defined
as not post-menopausal for 12 months or no previous surgical sterilization.
6. Inability to comply with medical therapy or follow-up.

4.0 Study Evaluations

<u>Evaluation During Study</u>
Every effort will be made to adhere to the schedule of events and all protocol requirements. Variations in schedule of events and other protocol requirements that do not affect the rights and safety of the patient will not be considered as deviations. Such variations may include laboratory assessments completed outside of schedule and occasional missed required research samples. Missed samples for correlative studies will not constitute protocol deviations.

<u>Evaluation Prior to Transplant (baseline).</u>
Standard work up for transplant as well as disease assessment is done prior to

study entry as part of diagnostic or routine pre-transplant evaluation.

Treatment Evaluations.

Blood will be drawn approximately on D+7, D+14, D+21, +28, before and approximately 1 week after the 3rd NK cell infusion, and then approximately at 3 months, 6 months and 12 months post-transplant, if feasible. Additional testing can be done at other timepoints as clinically indicated.

1. Peripheral blood immunophenotype panel/transplant panel.
2. Correlative studies may include:
    a. Evaluation for KIR expression on NK cells;
    b. NK cell function;
    c. Cytotoxicity of NK cells against patient's leukemia.

Excess NK cells produced during expansion beyond what is required to meet the current dose level will be delivered to the Dr. Dean Lee's laboratory at Nationwide Children's Hospital, Columbus, Ohio and to Dr. John Heymach's laboratory at MD Anderson for correlative studies. The samples will be labeled with name, date, and medical record number.

A total of 2cc/kg up to 60cc will be required, 5 (10cc) green top tubes and 1 (10cc) red top tube.

Dr. Dean Lee will collect data specific to the quantity, quality, phenotype, purity, and sterility at each step of manufacturing the NK cell products. Donor information related to expansion would include only the volume and cell count of the initial blood product received by the GMP facility, and HLA type, KIR type, and CMV status in order to correlate these variables with characteristics of the final product. The data will be used for a publication describing the real-life outcomes of manufacturing clinical-grade NK cells with this method.

Standard Post Transplant Evaluations

These evaluations follow our standard practice and are done to monitor engraftment and disease status:

1. Chimerism studies from peripheral blood performed on separated T-cells and myeloid cells.
2. At each visit, a physical examination and adverse event documentation including GvHD assessment.
3. Transplant Panel from the peripheral blood T-cell subsets, B-cell immune reconstitution, +/- NK cell studies.
4. Disease specific assessment as per standard of care and SCT&CT guidelines including bone marrow aspirate with cytogenetics and molecular studies if clinically indicated.

The following lab tests are to be performed as frequently as clinically indicated: CBC,

differential, platelets, SGPT, calcium, glucose, uric acid, magnesium, serum bilirubin, BUN and creatinine, serum protein, albumin, alkaline phosphatase, electrolytes, urinalysis, tacrolimus levels and CMV antigenemia.

5.0 Donor

To determine patient eligibility for this study, the donor must be previously identified. This process is carried out following standard procedures for donor eligibility. The donor must be 16 years of age or older and weigh at least 110 pounds.

This study involves two donations. One unit (approximately 500 mL or 3 ml/kg of recipient weight for pediatric patients) of peripheral blood will be drawn from the donor to start the NK cell expansion on aAPC for 14 days. The donor, parent(s) or guardian will provide consent as part of this research.

This process will occur approximately 16 days before the bone marrow stem cell infusion and after both patient and donor have provided consent.

The second donation is a bone marrow harvest of progenitor stem cells which will take place on the same day of the transplant. The process of bone marrow harvest is considered standard of care and the donor, parent(s), or guardian will sign a separate informed consent.

6.0 Treatment Plan

In this study the transplant day is referred to as day zero (D0), treatment plan activities prior or after D0 are denoted as day minus (D-) or day plus (D+).

<u>Approximately D-16. Collection and generation of NK cells</u>

The NK product on this trial will be manufactured in the Cell Therapy Laboratory GMP facility and all procedures will be validated according to their Laboratory Policy. The NK cell product is produced by first isolating peripheral blood mononuclear cells over Ficoll-Hypaque. NK cells are then propagated according to the procedures outlined in the CMC. Briefly, PBMC are co cultured with irradiated mIL21-aAPCs at a 2:1 (aAPC: PBMC) ratio. Cultures are maintained in culture media supplemented with 10% fetal calf serum and 100 IU/mL IL-2, with replenishment of media as needed. After 7 days, the cultures are restimulated with aAPCS at a 1:1 ratio. After one week, cultures may be depleted of residual CD3+ T-cells using the Miltenyi system (Miltenyi Biotec, Auburn, CA) using MACS colloidal super-paramagnetic CD3 MicroBeads conjugated with monoclonal mouse anti-human CD3 antibodies (Miltenyi Biotec) according to laboratory procedures. The Laboratory has well-established procedures for this system and is currently using this method for cell manufacturing under other INDs. Release criteria are according to the departmental guidelines.

The NK cell infusion is depleted of CD3+ cells by immunomagnetic separation as a method to eliminate T-cells in order to prevent development of GVHD.

The NK cell product will undergo lot release testing and cryopreservation in at least 2 aliquots per dose. Sterility testing will be partially completed at the time of cryopreservation, and will be repeated upon thawing of each product bag at the time of infusion.

Excess cells produced during expansion beyond what is required to meet the current dose level will be delivered to the Dr. Dean Lee's laboratory at Nationwide Children's Hospital, Columbus, Ohio for correlative studies. The samples will be labeled with name, date, and medical record number.

D-8 Admit/ Hydration- D-7 Melphalan Administration. Figure 1.

Melphalan will be administered after fludarabine on D- 7 at a dose of 140 mg/m^2 (4.6 mg/kg for patients less than 12 years old) in NS at a concentration of 1.5 mg/mL IV for one dose only. Patients weighing within 20% above their ideal body weight will be dosed according to actual body weight. Patients weighing more than 20% above their ideal body weight will be dosed according to the adjusted body weight. Formula to calculate adjusted body weight: Adjusted BW (Kg) = IBW + 0.5 (Actual body weight-IBW). Dosage will not be adjusted for pediatric patients weighing more than 20% above their ideal body weight. All patients will receive TBI 200 cGy which will be administrated on D-3. Figure 1.

Melphalan can be administered on D-8 at a dose of 100 mg/m2 in patients who won't be able to tolerate a full regimen based on comorbidities and /or age. Figure 2.

D-7 to D-4 Fludarabine Administration. Figure 1.

Fludarabine will be administered at the dose of 40 mg/m^2 in 100 ml of NS IV daily for four doses on days -7 to -4. Patients weighing within 20% above their ideal body weight will be dosed according to actual body weight. Patients weighing more than 20% above their ideal body weight will be dosed according to the adjusted body weight. Formula to calculate adjusted body weight: Adjusted BW (Kg) = IBW + 0.5 (Actual body weight-IBW). Dosage will not be adjusted for pediatric patients weighing more than 20% above their ideal body weight. Pediatric patients < 12 Kg will receive 1.3 mg/kg/day x 4 days.

D-2 or D-1: First NK infusion

Escalating doses of NK cells will be used in this study, starting at Dose level 2 ($1x10^5$ /kg) through dose level 6 ($1x10^8$/kg) CD3- CD56+ cells/kg. Dose level 1 ($1x10^4$/kg) will only be used if dose level 2 is not tolerated.

The Phase I study will find the maximum tolerated dose (MTD) which will be used in the Phase II study. The first infusion of expanded NK cells will be on day -2 or day -1, and the other 2 infusions will be using cryopreserved expanded NK cells. In case the

transplant is delayed, all infusions will be with frozen expanded NK cells.

The NK cell content will be based on total nucleated cell (TNC) count and flow cytometry assessment of CD56+ CD3- percentage. T-cell content will be based on total nucleated cell (TNC) count and flow cytometry assessment of CD3+ percentage. Patients will be infused cells according to the dosing scheme described below.
-The maximum volume of cell product infused is approximately 100 ml.
-The cells infused will be delivered on the basis of NK cells/kg recipient weight as defined by the current cohort.
-Total CD3+ T cells must be less than $1x10^5$/kg recipient weight for all cohorts. In order to get T cells < 10^5/kg we might need to do an additional T-cell depletion.
-If infusing the number of NK cells for the current cohort will result in delivering equal or higher than 10^5 CD3+ cells/kg recipient weight, the NK cell dose for infusion will be reduced to that of the highest cohort at which the infused CD3+ cells will be less than $1x10^5$/ kg recipient weight.
-Some donor NK cell expansions may not yield sufficient cells to reach the planned NK cell dose. If the target NK cell/kg recipient weight cannot be delivered, then the NK cell dose for infusion will be reduced to the highest cohort achievable. The patient data will be included on that cohort for statistical analysis, and the current dose level will enroll an additional subject.

Escalating doses of NK cells to be infused in the Phase I study as follows:
-Cohort 1 - 10^4/kg
-Cohort 2 - 10^5/kg
-Cohort 3- 10^6/kg
-Cohort 4 - 10^7/kg
-Cohort 5 - 3 x 10^7/kg
-Cohort 6 - 10^8/kg

For recipients who weigh > 100 kg, the NK cell dose will be determined as if their body weight is 100 kg.

NK cell infusion criteria:

To be able to receive the NK infusion(s) patients must meet the following requirements:

1. Off corticosteroids within prior 72 hour period (less than 0.5mg/kg prednisone or equivalent).
2. No active grade 2-4 aGVHD.
3. No uncontrolled infection or fever ≥ 38.5 C within 24 hours.
4. For day 28 dose, no grade 3 or greater nonhematologic organ toxicity.
5. Not requiring ventilator support or supplemental oxygen.

Premedication:

Patients are premedicated with diphenhydramine 25 mg intravenous piggy back (IVPB). Epinephrine and antihistamines should be available at the patient's bedside during the NK cell infusion to help treat any allergic reaction that might occur.

D-1 Rest

D0 Haploidentical bone marrow transplant

Infusion of a non T-cell depleted fresh graft. The target TNC number is $3x10^{8}$ cells/kg.

D+3 and D+4 Post Transplant Cyclophosphamide Administration with Mesna:

Patients will receive a dose of Mesna 10 mg/kg IVPB just prior to the first dose of cyclophosphamide, that will be repeated every 4 hours for a total of ten (10) doses. Patients will also receive ondansetron (or a comparable anti-emetic) and dexamethasone prior to each dose of Cyclophosphamide (Cy).

Patients will receive Cyclophosphamide at a dose of 50 mg/kg per dose. Patients weighing within 20% above their ideal body weight will be dosed according to actual body weight. Patients weighing more than 20% above their ideal body weight will be dosed according to the adjusted body weight. Formula to calculate adjusted body weight: Adjusted BW (Kg) = IBW + 0.5 (Actual body weight-IBW).

Hydration will be given per standard practice.

D+5 GvHD prophylaxis with Tacrolimus and Mycophenolate Mofetil per standard practice.Tacrolimus start 0.015 mg /kg (based on IBW (adjusted to a level 5-15ng/mL) IV daily continued PO for at least 4 months post transplant. The dose may be modified as clinically indicated. Mycophenolate mofetil (MMF) 15 mg/kg (max. 1000mg per dose, based on actual body weight) p.o. It is recommended start tapering at D+180, unless otherwise indicated, weekly over at least 3 weeks.

D+7 and D+28: Second and Third NK infusions

The second and third NK infusions will be performed on Day +7 (+/- 1 day) and Day +28-90 (when counts have recovered as indicated by ANC >/= 1.5 and no clinically significant GVHD), as described above.

G-CSF (Zarxio, filgrastim-sndz) and Supportive Care Antimicrobial prophylaxis as per standard practice.

D+7. G-CSF 5mcg/kg/day (rounded up to the nearest vial) s.q. once a day daily until neutrophil recovery.

Antimicrobial prophylaxis will be employed according to departmental standard practice.

Figure 1. Treatment schema using the myeloablative conditioning regimen.

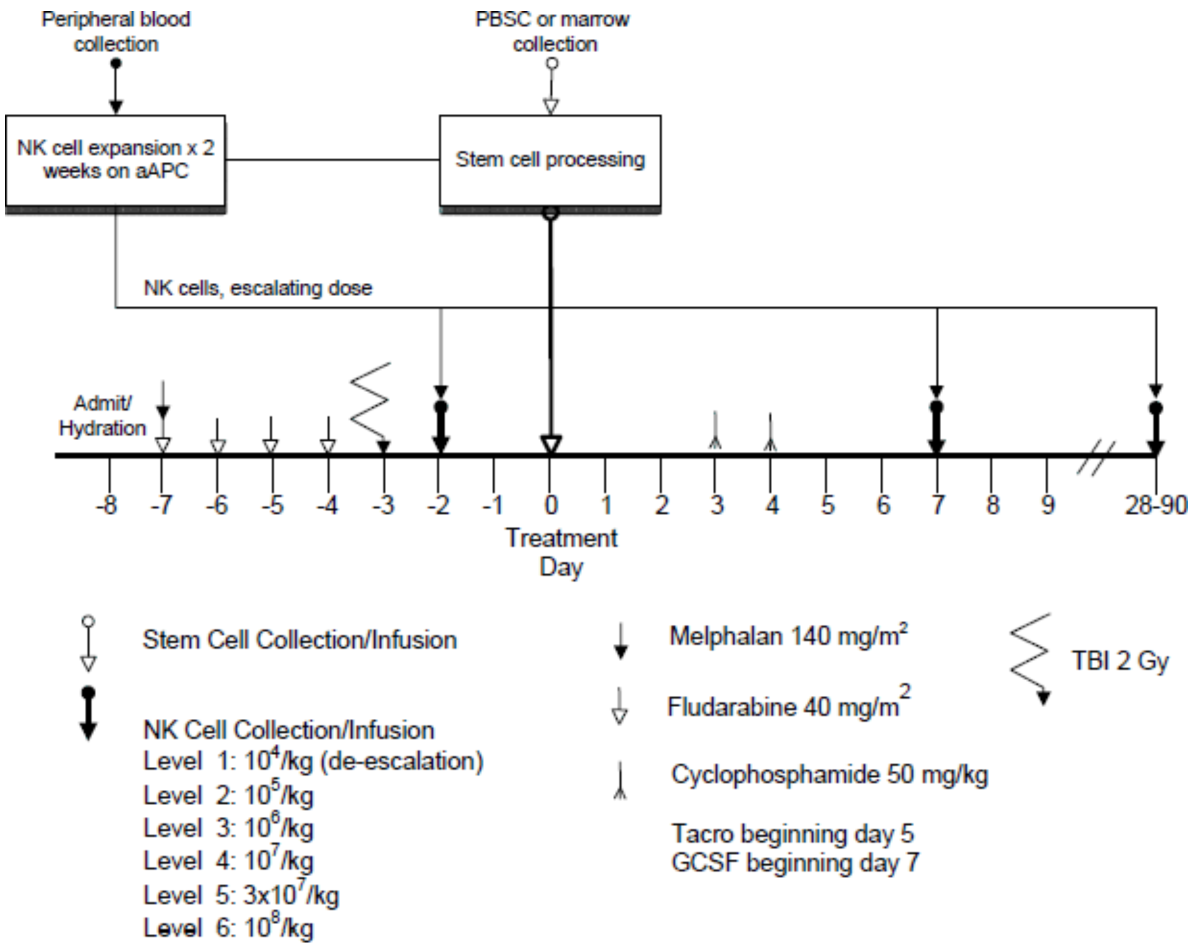

NK infusion days:
1st NK infusion: Day -2 or -1
2nd NK infusion: Day 7 (+/- 1 day)
3rd NK infusion: Day 28-90 (when counts have recovered as indicated by ANC >/= 1.5 and no clinically significant GVHD), as described above.

Figure 2. Treatment schema using the non-myeloablative conditioning regimen.

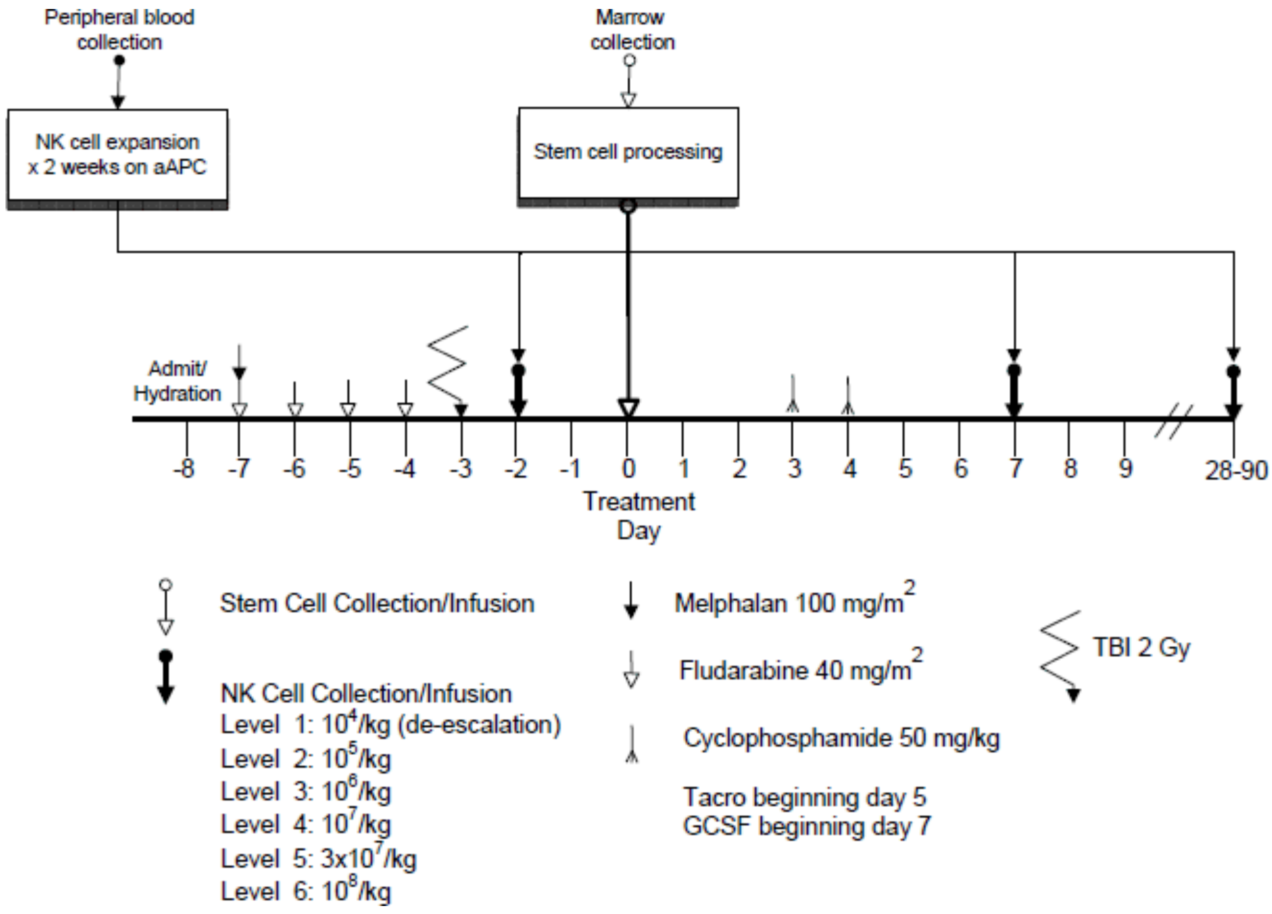

NK infusion days:
1st NK infusion: Day -2 or -1
2nd NK infusion: Day 7 (+/- 1 day)
3rd NK infusion: Day 28-90 (when counts have recovered as indicated by ANC >/= 1.5 and no clinically significant GVHD), as described above.

7.0 Study Criteria

Active treatment administration is defined from the first day of treatment administration as outlined in the treatment plan through the NK infusions.

Active treatment period is defined from the first day of treatment administration through 30 days post the third NK infusion.

Follow-up period is defined from 31 days post the third NK infusion until two years of treatment completion.

Engraftment is defined as the evidence of donor derived cells (more than 95%) by chimerism studies in the presence of neutrophil recovery by day 28 post stem cell infusion.

Other definitions used to assess engraftment:

Neutrophil recovery is defined as a sustained absolute neutrophil count (ANC) > 0.5 x 10^9/L for 3 consecutive days.

Engraftment date is the first day of three (3) consecutive days that the ANC exceeds 0.5 x10^9/L.

Delayed engraftment is defined as the evidence of engraftment beyond day 28 post SC infusion achieved after the administration of therapeutic (high dose) hematopoietic growth factors.

Primary Graft failure is defined as failure to achieve an ANC > 0.5 x 10^9/L for 3 consecutive days by day 28 post SC infusion, with no evidence of donor derived cells by bone marrow chimerism studies and no evidence of persistent or relapsing disease.

Secondary graft failure is defined as a sustained declined of ANC <0.5 x 10^9/L for 3 consecutive days after initial documented engraftment with no evidence of disease progression.

Autologous reconstitution is defined by the presence of ANC > 0.5 x 10^9/L without evidence of donor-derive cells by bone marrow chimerism studies. This can occur at initial engraftment or later after initial engraftment has been documented.

Disease Response as per CIBMTR criteria.

A. For AML/MDS:

Complete remission (CR):
BM < 5% blasts (absence of blasts with Auer rods).
ANC > 1000/ul.
Platelet count >100 x 10e9/ul (independent of red cell transfusions).
Absence of extramedullary disease.

Marrow CR (CRi) (incomplete hematologic recovery).
BM < 5% blasts (absence of blasts with Auer rods).
ANC < 1000/ul or Platelet count <100 x 10e9/ul.
Absence of extramedullary disease.

No Response (NR) or Disease Progression
BM > 5% leukemia blasts
Persistent presence of blasts in peripheral blood.
Presence of extramedullary disease.

B. For CML:

Cytogenetic Response
Complete: No Ph positive metaphases.
Major: 0-35% Ph positive metaphases.
Partial: 1-34% Ph positive metaphases.
Minor: 35-90% Ph positive metaphases.

Complete Hematologic Response
Complete normalization of peripheral blood counts with leukocyte count <10x10^9/L.
Platelet count <450x10^9/L.
No immature cells in peripheral blood.

No signs and symptoms of disease with disappearance of palpable splenomegaly.

Partial Hematologic Response
Same as complete hematologic response, except for:
Presence of immature cells.
Platelet count < 50% of the pretreatment count, but >450x10^9/L..
Persistent splenomegaly, but < 50% of the pretreatment extent.

Molecular Response:
Complete molecular response: BCR-ABL mRNA undetectable by RT-PCR.
Major molecular response equal or more 3-log reduction of BCR-ABL mRNA.

Non-relapse mortality (NRM) is defined as death from any cause other than relapse disease.

Disease free survival (DFS) is defined as the interval between day of transplant and day of death or disease progression.

Overall Survival (OS) is defined as the interval between day of transplant and day of death.

Off study criteria

1. Inability to infuse the NK cell product due to product contamination or insufficient cell dose.
2. Disease progression requiring further treatment.
3. Prolonged cytopenia requiring treatment beyond GCSF.
4. Unexpected pattern of toxicity.
5. Patient withdrawal of the informed consent.
6. Patient is noncompliant with treatment schema.
7. After two years of treatment completion.

8.0 Adverse Events and Reporting Requirements

PDMs/CORe will be used as the electronic case report form (eCRF) for this protocol and protocol specific data including adverse events will be entered into PDMs/CORe. The Investigator or physician designee is responsible for verifying and providing source documentation for all adverse events and assigning the attribution for each event for all subjects enrolled on the trial. Only adverse events with a direct relationship to the NK cell infusion will be captured in PDMS/CORE.

Assessment of the Adverse Events Attribution.
The investigational component of the treatment plan of this study is the NK cell infusion. The preparative regimens used in this study are considered standard of care and their associated adverse events are well known. Therefore, for the purpose of this study when, in the presence of an adverse event which a direct relationship to the NK cell

infusion is suspected, the event will be attributed to the NK cell infusion.

Events known to be caused by the preparative regimens and their direct consequences, as well as those events known to be related to drugs used for the prophylaxis and treatment of GvHD, infections, and supportive treatment will be scored as unrelated to the NK cell infusion.

The principal investigator will be the final arbiter in determining the attribution of the event.

Assessment of the Adverse Events Severity.

The severity of the adverse events (AEs) will be graded according to the Common Terminology Criteria v4.0 (CTCAE) from the start of the day of the first NK cell infusion to Day 70 post-transplant. Events not included in the CTCAE chart will be scored as follows:

<u>General grading:</u>

Grade 1: Mild: discomfort present with no disruption of daily activity, no treatment required beyond prophylaxis.

Grade 2: Moderate: discomfort present with some disruption of daily activity, require treatment.

Grade 3: Severe: discomfort that interrupts normal daily activity, not responding to first line treatment.

Grade 4: Life Threatening: discomfort that represents immediate risk of death.

Expected Adverse Events possibly associated with infusion of allogeneic NK cells:

1. Acute adverse events :

Events lasting less than 24 hours:

Grade I chills
Grade I cough
Grade I or II angioedema
Grade I or II dyspnea
Grade I or II hypotension
Grade I or II tachycardia
Grade I or II headache

Events lasting less than 48 hours:

Grade I or II fatigue
Grade I or II neuropathic pain
Grade I or II vomiting
Grade I or II SGPT changes
Grade I or II hypoalbuminemia
Grade I or II hypocalcemia
Grade I or II fever
Grade I or II pruritus
Grade I rash

Grade I or II lymphopenia
Grade I or II neutropenia
Grade I or II leukopenia
Grade I or II cytokine release/acute infusion reaction

2. Events lasting less than 72 hours:
Grade I or II nausea
Tumor Lysis Syndrome

List of most common expected adverse events related to Stem Cell transplantation and Preparative Regimen.

1. Infections in the presence or absence of neutropenia: fungal, bacterial and or viral infections.
2. Fever: Non-neutropenic or neutropenic without infection
3. Acute graft versus host disease (aGvHD): most commonly manifested by skin rash, diarrhea and abnormal liver function tests could also present with some degree of fever, upper gastrointestinal symptoms (nausea and vomiting) mucositis and eye dryness.
4. Gastrointestinal (GI tract): the GI tract manifestations could be not only due to direct damage from the preparative regiment but also be a manifestation of GvHD or infections. Therefore, the time course and its presentation are crucial when assessing these as adverse events. Nausea/vomiting, mucositis, diarrhea when presented within first 7 to 10 days most likely will be related to the preparative regimen.
5. Skin rash: not related to GvHD could be caused by chemotherapy used for the preparative regimen or antibiotics used a supportive treatment.
6. Transaminitis: liver function test elevation.
7. Pulmonary events: not related to CHF most likely caused by drug injury or infection. These could present with a pneumonitis pattern manifested with shortness of breath, pulmonary infiltrates on chest radiograph, sometimes accompanied by fever and cough and progress to acute respiratory insufficiency and a diffuse bilateral alveolar pattern.
8. Cytokine Storm/ engraftment syndrome: most likely caused by released cytokines.
9. Hemorrhagic cystitis: not related to chemotherapy agents used in the proposed preparative regimen is most likely caused by viral infection.
10. Thrombotic thrombocytopenic purpura (TTP).
11. Veno-occlusive Disease of the Liver (VOD): could be caused by busulfan. Some antimicrobial agents have been also incriminated in its development.
12. Fluid overload due to hydration required for conditioning regimen, blood product transfusions and or IV alimentation
13. Graft failure.
14. Chronic GvHD.
15. For the purpose of this study the following events would not be considered adverse events and would not be recorded in the database:
    1. Flu-like symptoms not associated with infection
    2. Abnormal laboratory findings considered associated to the original disease

3. Isolated changes in laboratory parameters such as electrolyte, magnesium and metabolic imbalances, uric acid changes, elevations of ALT, AST, LDH and alkaline phosphatase.

Adverse events data collection.

From the the first NK infusion up to Day +70 post-transplant, only adverse events considered unexpected and related will be collected. The data will reflect the onset and resolution date and maximum grade. Beyond this point late complications post transplant such as chronic GvHD might be recorded up to a year from the time of their awareness with no grade or resolution date.

Intermittent events should be labeled as such and followed until resolution.

If a patient is taken off study while an event is still ongoing, this will be followed until resolution unless another therapy is initiated. Pre-existing medical conditions will be recorded only if an exacerbation occurs during the active treatment period. Co-morbid events will not be scored separately.

Concurrent medication

As stated in the treatment plan, patients treated on this protocol will required supportive care treatment (concurrent medication). These medications are considered standard of care and have no scientific contributions to the protocol, therefore no data will be captured on the various medications needed or their sides effects.

AE and Protocol Deviations Reporting Requirements.

Adverse events will be reported accordingly to MDACC (HSRM chapter 15.001) policy and procedures.This study will be conducted in compliance however in the event of any protocol deviations or violations these will be reported accordingly to MDACC (HSRM chapter 25).

Serious Adverse Event Reporting (SAE)

An adverse event or suspected adverse reaction is considered “serious” if, in the view of either the investigator or the sponsor, it results in any of the following outcomes:

- Death
- A life-threatening adverse drug experience – any adverse experience that places the patient, in the view of the initial reporter, at immediate risk of death from the adverse experience as it occurred. It does not include an adverse experience that, had it occurred in a more severe form, might have caused death.
- Inpatient hospitalization or prolongation of existing hospitalization
- A persistent or significant incapacity or substantial disruption of the ability to conduct normal life functions.
- A congenital anomaly/birth defect.

Important medical events that may not result in death, be life-threatening, or require hospitalization may be considered a serious adverse drug experience when, based upon appropriate medical judgment, they may jeopardize the patient or subject and may require medical or surgical intervention to prevent one of the outcomes listed in this definition. Examples of such medical events include allergic bronchospasm requiring intensive treatment in an emergency room or at home, blood dyscrasias or convulsions that do not result in inpatient hospitalization, or the development of drug dependency or drug abuse (21 CFR 312.32).

- **Important medical events as defined above, may also be considered serious adverse events. Any important medical event can and should be reported as an SAE if deemed appropriate by the Principal Investigator or the IND Sponsor, IND Office.**

- All events occurring during the conduct of a protocol and meeting the definition of a SAE must be reported to the IRB in accordance with the timeframes and procedures outlined in "The University of Texas M. D. Anderson Cancer Center Institutional Review Board Policy for Investigators on Reporting Unanticipated Adverse Events for Drugs and Devices". Unless stated otherwise in the protocol, all SAEs, expected or unexpected, must be reported to the IND Office, regardless of attribution (within 5 working days of knowledge of the event).

- **All life-threatening or fatal events,** that are unexpected, and related to the study drug, must have a written report submitted within **24 hours** (next working day) of knowledge of the event to the Safety Project Manager in the IND Office.

- **Unless otherwise noted, the electronic SAE application (eSAE) will be utilized for safety reporting to the IND Office and MDACC IRB.**

- **Serious adverse events will be captured from the time of the first protocol-specific intervention, until 30 days after the last dose of drug, unless the participant withdraws consent. Serious adverse events must be followed until clinical recovery is complete and laboratory tests have returned to baseline, progression of the event has stabilized, or there has been acceptable resolution of the event.**

- **Additionally, any serious adverse events that occur after the 30 day time period that are related to the study treatment must be reported to the IND Office. This may include the development of a secondary malignancy.**

Reporting to FDA:

- Serious adverse events will be forwarded to FDA by the IND Sponsor (Safety Project Manager IND Office) according to 21 CFR 312.32.

It is the responsibility of the PI and the research team to ensure serious adverse

events are reported according to the Code of Federal Regulations, Good Clinical Practices, the protocol guidelines, the sponsor's guidelines, and Institutional Review Board policy.

9.0 Statistical Considerations

Background

This is a phase I-II trial of natural killer (NK) cells used with the reduced intensity preparative regimen fludarabine + melphalan +/-TBI for allogeneic stem cell transplant (SCT) in high risk myeloid malignancies, defined as either at high risk of relapse while in complete remission (CR), or not in first CR. For the purpose of dose-finding, "toxicity" will be defined as any of the events (i) death, (ii) grade 3-4 infusion reaction, (iii) grade 4 organ toxicity (not including mucositis or myelosuppression) (iv) graft failure, or (v) severe (grade 3 or 4) graft-versus-host disease (GVHD) within the 72-day window occurring between the time of the first NK cell dose and day 70 post SCT. Patient outcome will be the time of occurrence of toxicity or, if toxicity has not yet occurred by an observation time prior to day +70, the outcome will be the patient's follow up time (starting on the day of the first NK infusion) without toxicity. Additional outcomes include 100-day treatment related mortality (TRM100), defined as death due to any cause without disease recurrence within 100 days post SCT, and overall survival (OS) time, relapse-free survival (RFS) time, and the times to engraftment and graft failure, each subject to right-censoring. Under the original study design, dose-finding was to be carried out using the time-to-event (TiTE) continual reassessment method (CRM) of Cheung and Chappell [(27)]. The seven NK cell doses to be studied were d = number of NK cells = 10E4, 10E5, 10E6, 10E7, 3X10E7, 10E8, and 3X10E8. The TiTE CRM was to be applied with cohorts of size 2, starting at d = 10E5 (the second NK cell dose level), not skipping a dose level when escalating, target Pr(toxicity by day 70) = 0.50, and maximum sample size 30. There was at least a 96-hour interval between patient entry within the first cohort to be able to observe for severe infusion-related toxicity. If a new patient arrives to be treated at a time when either 1 or 2 of the patients in the previous cohort have been treated but not yet fully evaluated, then the new patient will be treated at one dose level below the current recommended level, up to a maximum of 3 new patients.

The TiTE CRM design was to be implemented by Roland Bassett or his designee in the Department of Biostatistics using a specialized computer program, based on a repeatedly updated data file containing patient accession number, NK cell dose, transplant date, and outcome (T,C) and outcome date (either date of T or last visit date if no T) for each patient, provided by designated personnel in the Department of Stem Cell Transplantation and Cellular Therapy. For Phase II safety monitoring, the method of Thall and Sung (28), based on an historical rate of 35%, will be applied to monitor the TRM100 probability at any dose. All adverse events will be tabulated by dose, and the fitted dose-toxicity curve will be summarized. The distributions of all event time outcomes will be estimated using Kaplan-Meier plots. [29]

Overview

This is a phase I-II trial of natural killer (NK) cells used with the reduced intensity preparative regimen fludarabine + melphalan +/- TBI for allogeneic stem cell transplant (SCT) in high risk myeloid malignancies, defined as either at high risk of relapse while in complete remission (CR), or not in first CR. The NK cells will be given at the prescribed dose three times: day -2 or day -1 pre-SCT and at days 7 and 28 post SCT.

Phase I

NK Cell Dose-Finding
The original dose-finding algorithm is described in the following three paragraphs:

For the purpose of dose-finding, “toxicity” will be defined as any of the events (i) death, (ii) grade 3-4 infusion reaction, (iii) grade 4 organ toxicity (not including mucositis or myelosuppression) (iv) graft failure, or (v) severe (grade 3 or 4) graft-versus-host disease (GVHD) occurring within 72 days of the first NK cell infusion on day -2 or day -1, that is, by day 70 post SCT. Consequently, the time window for evaluating toxicity will be of length 72 days, from the day of the first NK cell infusion to day 70. Patient outcome will be the time of occurrence of toxicity or, if toxicity has not yet occurred by an observation time prior to day 70 post-transplant, the outcome will be the patient’s follow up time without toxicity. Denoting the time of follow up or toxicity by T, and the right-censoring indicator C=1 if T is a follow up time without toxicity and C=0 if T is the time of observed toxicity, each patient’s data will consist of the pair (T,C), with T no larger than 72 days. A patient’s outcome (T,C) will be considered “fully evaluated” if either C= 0 (toxicity has occurred at some time up to day 70 post-transplant) or (T,C) = (72,1), which says that no toxicity has occurred by day 70. Patients age 2-17 years old may be enrolled after at least 4 adults (ages 18-65 years old) have been treated without toxicity. Toxicity data will be submitted for FDA review and approval prior to expanding access to subjects age 2-17 years old. Additional outcomes include 100-day treatment related mortality (TRM100), defined as death due to any cause without disease recurrence within 100 days post SCT, and possibly right-censored overall survival time, relapse-free survival time, and the times to engraftment and graft failure.

Dose-finding will be carried out using the time-to-event (TiTE) continual reassessment method (CRM) of Cheung and Chappell (27). Denoting exponentiation by “E,” the six NK cell per-administration doses (PADs) to be studied are number of NK cells = 10E4, 10E5, 10E6, 10E7, 3x10E7, and 10E8. The TiTE CRM will be applied with cohorts of size 2, starting at 10E5 (the second lowest NK cell PAD level), not skipping a dose level when escalating, target Pr(toxicity by day 70 post-transplant) = 0.50, and maximum sample size 30. To avoid aggressive/unsafe escalation early in the trial, the design will proceed in two stages. In Stage 1, the minimum waiting time between dose cohorts is 49 days from the first NK infusion. The first cohort of two patients will be enrolled at d = 10E5 NK cells. If no toxicity is observed, then two new patients (the second cohort) will be enrolled at d = 10E6 NK cells. If no toxicities are observed among the previously accrued patients, then escalation will proceed in cohorts of size 2 to subsequently higher doses. If a new patient arrives to be treated at a time when either 1 or 2 of the patients in the previous cohort have been treated but not yet fully evaluated, then the

new patient will be treated at one dose level below the current recommended level, up to a maximum of 3 new patients. Stage 2 begins when the first toxicity occurs, and thereafter the cohort size will be 1 with the TiTE-CRM used to choose each new patient's dose, using the fixed target 0.50 for Pr(toxicity within 72 days | dose). The "optimal" dose is defined as that for which the posterior mean of Pr(toxicity within 72 days | dose) given the current data is closest to 0.50. Each subject will be followed for the entire 72-day period for toxicity evaluation. Once a DLT occurs at any time in the 72-day window for any subject, new subjects will be assigned to the optimal dose. Additional safety restrictions applied during the trials are (1) an untried dose may not be skipped when escalating and (2) escalation may not be done immediately after a toxic outcome (i.e., incoherent escalation). The skeleton corresponding to dose levels 1-7 that is the basis for the assumed TiTE CRM model is (0.30, 0.35, 0.42, 0.50, 0.60, 0.68, 0.75) .

The TiTE CRM design will be implemented by Roland Bassett or his designee in the Department of Biostatistics using a specialized computer program, based on a repeatedly updated data file containing patient accession number, NK cell dose, transplant date, and outcome (T,C) and outcome date (either date of T or last visit date if no T) for each patient, provided by designated personnel in the Department of Stem Cell Transplantation and Cellular Therapy.

June 2016 Update: The study team used a different algorithm to perform dose-finding than the one specified. Patients were treated in cohorts of size 2, with escalation after every two patients. The top level reached was dose level 6, 10E8. No patients experienced DLT, and the top level, 3X10E8 is unable to be manufactured. The team thus plans to move to the Phase II portion of the study. The statistical considerations for the Phase II portion remain unchanged.

After the conclusion of the Phase I portion of the design, the study team plans to publish the safety and efficacy results to date. The analysis will include assessing overall and progression-free survival and compare these between patients in this study and patients treated on M.D. Anderson protocol 2009-0266, an ongoing protocol of stem cell transplant in haploidentical patients who were treated with a similar regimen but without NK cells. As this is not a randomized comparison, and patients were treated at different times, statistical calculations of p-values and confidence intervals will be provided on an descriptive basis only and have no inferential content.

Phase II

Sample Size and Estimation

Once an optimal dose NK cell per-administration PAD has been determined in phase I, denote it PAD*, an additional 32 patients will be enrolled in phase II. The 13 patients treated in phase I will be counted toward the phase II sample, so the total maximum phase II sample will be 32 + 13 = 45. All patients will be followed for 2 years. The goals of phase II will be to estimate relapse-free survival (RFS), overall survival (OS), overall transplant related mortality, the times to platelet and neutrophil engraftment, and the

probability of TRM100 at PAD*. For example, with a total of 2 patients treated at the PAD* in phase I and 32 in phase II, and 16/34 TRM100 events are observed, starting with a beta(.5, .5) prior, a posterior 95% credible interval for Pr(TRM100 at PAD*) would be 0.310 - 0.634.

Monitoring TRM100

While "toxicity" is defined as any of the several adverse events listed above within 70 days post-transplant, there is still the concern that the TRM100 rate at a given NK cell PAD may be too high. Using the monitoring method of Thall and Sung,[28] based on an historical rate of 35%, we assume that the 100-day TRM probability at any PAD, denoted by q(TRM100 | PAD), follows a beta(0.35, 0.65) prior.

The safety rule is to stop treatment at dose d due to excessive mortality if P{ q(TRM100 | PAD) > 0.35 | data from patients treated at dose d} > 0.975. Monitoring in cohorts of size 5, this implies that treatment at dose d will be stopped if

[# TRM100 observed at dose d] / [# evaluated by day 100 at dose d} ≥
5/5, 7/10, 10/15, 12/20, 14/25, 16/30, 19/35, 21/40

If enrollment is stopped at dose d by this rule, then it will be stopped at all higher dose levels, and this rule will supersede the TiTE CRM.

Data Analysis

All adverse events will be tabulated by dose, and the fitted dose-toxicity curve will be summarized. The distributions of time-to-event outcomes, including RFS/OS, and times to platelet and neutrophil engraftment will be estimated separately by dose using the Kaplan-Meier method,[29] and distributions will be compared using log-rank tests.

Table 1. Operating characteristics of the TiTE CRM dose-finding design. A maximum sample size of 30 patients and accrual rate of 1 patient per month are assumed, starting at dose level 2, with cohort size 2, and toxicity target 0.50 for Prob(toxicity within 70 days of SCT).

		**1**	**2**	**3**	**4**	**5**	**6**	**7**
	**# NK cells**	10E4	10E5	10E6	10E7	3X10E7	10E8	3X10E8
**Scenario**								
1	True Prob(Tox)	0.30	0.35	0.42	0.50	0.60	0.68	0.75
	% Selected	3.3%	6.0%	22.7%	42.5%	21.5%	4.0%	0.0%
	Average N Patients Treated	3.5	4.7	6.2	8.3	5.5	1.6	0.4
	Average N Patients w/Tox	1.1	1.6	2.6	4.1	3.3	1.1	0.3
2	True Prob(Tox)	0.25	0.30	0.36	0.47	0.53	0.56	0.60
	% Selected	0.3%	2.6%	12.9%	34.3%	31.9%	12.6%	5.4%
	Average N Patients Treated	2.0	3.8	5.2	8.0	6.4	3.0	1.6
	Average N Patients w/Tox	0.5	1.1	1.8	3.8	3.4	1.7	0.9

3	True Prob(Tox)	0.20	0.25	0.30	0.35	0.40	0.45	0.50
	% Selected	0%	0.4%	2.1%	10.9%	28.3%	29.3%	29.0%
	Average N Patients Treated	1.2	2.9	3.1	4.9	7.0	5.8	5.1
	Average N Patients w/Tox	0.2	0.7	0.9	1.7	2.8	2.6	2.5
4	True Prob(Tox)	0.60	0.65	0.70	0.75	0.80	0.85	0.90
	% Selected	90.8%	5.2%	3.2%	0.7%	0.1%	0%	0%
	Average N Patients Treated	22.1	4.3	2.2	1.0	0.3	0.0	0.0
	Average N Patients w/Tox	13.4	2.8	1.5	0.8	0.2	0.0	0.0
5	True Prob(Tox)	0.50	0.60	0.70	0.80	0.90	0.95	0.98
	% Selected	74.0%	21.0%	4.9%	0.1%	0%	0%	0%
	Average N Patients Treated	18.8	6.6	3.3	1.2	0.2	0.0	0.0
	Average N Patients w/Tox	9.3	4.0	2.3	0.9	0.2	0.0	0.0

Table 2. Operating characteristics of the safety rule for monitoring the probability of TRM100 = treatment-related mortality by day 100 at each dose, assuming a maximum of 45 patients at any given dose. The historical TRM100 rate is 35%.

		**Sample Size Quartiles**		
**True TRM100 Rate**	**Probability of Stopping Early**	**25th**	**50th (Median)**	**75th**
20%	0.001	45	45	45
25%	0.006	45	45	45
30%	0.022	45	45	45
35%	0.069	45	45	45
40%	0.174	45	45	45
45%	0.340	30	45	45
50%	0.567	20	35	45
55%	0.786	10	20	40
60%	0.911	10	20	25

A retrospective case matched comparison will be performed by CIBMTR using data from study patients already reported to CIBMTR. Controls will be obtained from CIBMTR database also at 3-4 for each patient treated on this study.

10.0 Drug Information

Melphalan (Alkeran™)

Melphalan is an alkylating agent with cell cycle nonspecific cytotoxic effects on tumor cells.
Mechanism of Action: Inhibits DNA replication and transcription of RNA ultimately disrupting nucleic acid function.
Dosing Information: The usual dose for conditioning in stem cell transplantation is 100-200 mg/m2 intravenously.
Known Side Effects: Hematologic: The most common adverse effect is bone marrow

suppression. Leukopenia and thrombocytopenia are the major dose limiting side effects. GI toxicity is frequent with higher doses and includes nausea and vomiting, stomatitis, abdominal cramping and diarrhea. Other adverse effects: Pulmonary fibrosis and infiltrates, amenorrhea, alopecia, sterility, and inappropriate ADH secretion. Hypersensitivity reactions: Acute hypersensitivity reactions have been reported including urticaria, pruritus, edema, tachycardia, bronchospasm and anaphylaxis (reported in 2.4% of patients receiving melphalan for myeloma). These patients respond to antihistamine and corticosteroid therapy.
Special Precautions: None

Fludarabine (Fludara™)

Fludarabine is a fluorinated nucleoside analog.
Mechanism of Action: After phosphorylation to fluoro-ara-ATP the drug appears to incorporate into DNA and inhibit DNA polymerase alpha, ribonucleotide reductase and DNA primase, thus inhibiting DNA synthesis.
Human Safety and Pharmacology: The half-life of the activated compound is approximately 10 hours, but the pharmacology is incompletely understood. Excretion is impaired in patients with impaired renal function.
Known Side Effects: pancytopenia, immunosuppression, autoimmune hemolytic anemia have (rarely) been reported, and recurred when patients were retreated with the drug. GI toxicity: Nausea, vomiting, anorexia, weakness. CNS toxicity: agitation, visual disturbances, confusion, coma, peripheral neuropathies. With high dose use confusion, blindness, coma and death have been reported.
Special Precautions: Fludarabine should be handled by trained personnel using procedures for proper handling. The use of gloves and protective glasses is recommended to avoid exposure upon accidental spillage.

Cyclophosphamide (Cytoxan™)

Cyclophosphamide is an alkylating agent.
Mechanism of Action: Cyclophosphamide prevents cell division by cross-linking DNA strands and decreasing DNA synthesis. It is cell cycle phase non-specific. Cyclophosphamide also possesses potent immunosuppressive properties. It is a pro-drug metabolized by the liver to active metabolites.
Human Safety and Pharmacology: The Distribution (Vd) is 0.48-0.71 L/kg; crosses placenta; crosses into CSF. Protein binding is 10-60%. Hepatically metabolized to active metabolites acrolein, 4-aldophosphamide, 4-hydroperoxycyclophosphamide, and nor-nitrogen mustard. Bioavailability is >75%. Half-life elimination is 3-12 hours. Excretion in urine (<30% as unchanged drug, 85% to 90% as metabolite).
Known Side Effects: Hematologic: Leukopenia, anemia, alopecia. GI: Nausea, vomiting, increased AST, ALT, mucositis, diarrhea. Neurologic: Headache, dizziness. Cardiovascular: Cardiomyopathy, non-specific ST changes on EKG. At doses greater than 200mg/kg, Cy can cause fatal myocardial necrosis with clinical heart failure. Renal: Hemorrhagic cystitis, SIADH, fluid retention. Cy has anti-diuretic effect usually

counteracted by furosemide administration. GU: Hemorrhagic cystitis. Hematuria is not uncommon at this dose level, but is usually not symptomatic or severe unless there is inadequate diuresis. An occasional patient will get severe cystitis despite prophylactic measures. Other: teratogenic, may cause secondary neoplasms, anaphylaxis (rare). Special Precautions: As for other antineoplastic agents, Cyclophosphamide should be handled by trained personnel using procedures for proper handling. The use of gloves and protective glasses is recommended to avoid exposure upon accidental spillage.

Mesna (sodium -2-mercapto ethane sulphonate)

Mesna is a prophylactic agent used to prevent hemorrhagic cystitis induced by the oxazophosphorines (cyclophosphamide and ifosfamide). It has no intrinsic cytotoxicity and no antagonistic effects on chemotherapy. Mesna binds with acrolein, the urotoxic metabolite produced by the oxazophosphorines, to produce a non-toxic thioether and slows the rate of acrolein formation by combining with 4-hydroxy metabolites of oxazophosphorines. At the doses used for uroprotection, mesna is virtually non-toxic. However, adverse effects which may be attributable to mesna include nausea and vomiting, diarrhea, abdominal pain, altered taste, rash, urticaria, headache, joint or limb pain, hypotension and fatigue.

11.0 References

1. Bayraktar UD, Champlin RE, Ciurea SO. Progress in haploidentical stem cell transplantation. Biol Blood Marrow Transplant. 2011 Aug 8. [Epub ahead of print].

2. Ciurea SO, Saliba R, Rondon G, et al. Reduced-Intensity Conditioning Using Fludarabine, Melphalan and Thiotepa for Adult Patients Undergoing Haploidentical Stem-Cell Transplantation. Bone Marrow Transplant. 2010;45:429-36.

3. Luznik L, Jalla S, Engstrom LW, Iannone R, Fuchs EJ. Durable engraftment of major histocompatibility complex-incompatible cells after nonmyeloablative conditioning with fludarabine, low-dose total body irradiation, and posttransplantation cyclophosphamide. Blood. 2001;98:3456-3464.

4. Brunstein C, Fuchs EJ, Carter SL, et al. Alternative donor transplantation after reduced-intensity conditioning: results of parallel phase 2 trials using partially HLA-mismatched related bone mwrrow or unrelated double umbilical cord blood grafts. Blood. 2011;118(2):282-288.

5. Ciurea SO, Mulanovich V, Saliba RM, et al. Improved Early Outcomes with T-cell Replete Graft Compared with T-cell Depleted Haploidentical Hematopoietic Stem Cell Transplantation. Biol Blood Marrow Transplant. 2012 July 11. [Epub aheadof print].

6. Freud AG, Caligiuri MA. Human natural killer cell development. Immunol Rev. 2006;214:56-72.

7. Caligiuri MA. Human natural killer cells. Blood. 2008;112(3):461-9.

8. Farhan S, Lee D, Champlin RE, Ciurea SO. NK cell therapy: targeting disease relapse after hematopoietic stem cell transplantation. Immunotherapy. 2012;3:305-313.

9. Lanier LL. Follow the leader: NK cell receptors for classical and nonclassical MHC class I. Cell. 1998;92(6):705-707.

10. Karre K, Ljunggren HG, Piontek G, Kiessling R. Selectiove rejection of H-2-deficient lymphoma variants suggests alternative immune defence strategy. Nature. 1986;319:675-678.

11. Leung W, Iyengar R, Turner V, et al. Determinants of antileukemia effects of alloreactive NK cells. J Immunol. 2004;172:644-650.

12. Malmberg KJ, Schaffer M, Ringden O, Remberger M, Ljunggren HG. KIR-ligand mismatch in allogeneic hematopoietic stem cell transplantation. Mol Immunol. 2005;42(4):531-4.

13. Murphy WJ, Parham P, Miller JS. NK cells--from bench to clinic. Biol Blood Marrow Transplant. 2012;18(1 Suppl):S2-7.

14. Ruggeri L, Capanni M, et al. Effectiveness of donor natural killer cell alloreactivity in mismatched hematopoietic transplants. Science. 2002;295(5562):2097-2100.

15. Gill S, Olson JA, Negrin RS. Natural killer cells in allogeneic transplantation: effect on engraftment, graft-versus-tumor, and graft-versus-host responses. Biol Blood Marrow Transplant. 2009;15(7):765-76.

16. Kim DH, Sohn SK, Lee NY, et al. Transplantation with higher dose of natural killer cells associated with better outcomes in terms of non-relapse mortality and infectious events after allogeneic peripheral blood stem cell transplantation from HLA-matched sibling donors. Eur J Hematol. 2005;75(4):299-308.

17. Rizzieri DA, Storms R, Chen DF, et al. Natural killer cell-enriched donor lymphocyte infusions from a 3-6/6 HLA matched family member following nonmyeloablative allogeneic stem cell transplantation. Biol Blood Marrow Transplant. 2010;16:1107-1114.

18. Champlin RE, Lee DA, Fernandez-Vina M, et al. Alloreactive NK cells to augment antileukemia cytotoxicity with allogeneic stem cell transplantation for AML/MDS: A phase I study. Blood. 2010;116:489a.

19. Miller JS, Soignier Y, et al. Successful adoptive transfer and in vivo expansion of human haploidentical NK cells in patients with cancer. Blood. 2005;105(8):3051-7.

20. Denman C, Senyukov, VV, Somanchi SS, et al. Membrane-bound IL-21 promotes sustained *ex vivo* proliferation of human natural killer cells. PloS One. 2012;7:1-13.

21. Shilling HG, McQueen KL, Cheng NW, Shizuru JA, Negrin RS, Parham P. Reconstitution of NK cell receptor repertoire following HLA-matched hematopoietic cell transplantation. Blood. 2003;101:3730-40.

22. Chklovskaia E, Nowbakht P, Nissen C, et al. Reconstitution of dendritic and natural killer-cell subsets after allogeneic stem cell transplantation: effects of endogenous flt3 ligand. Blood. 2004;103:3860-8.

23. Nguyen S, Dhedin N, Vernant JP, et al NK-cell reconstitution after haploidentical hematopoietic stem-cell transplantations: immaturity of NK cells and inhibitory effect of NKG2A override GvL effect. Blood. 2005;105:4135-4142.

24. Wang H, Grzywacz B, Sukovich D, et al. The unexpected effect of cyclopsporin A on CD56+CD16- natural killer cell subpopulation. Blood. 2007;110:1530-9.

25. Savani BN, Mielke S, Adams S, et al. Rapid natural killer cell recovery determines outcome after T-cell-depleted HLA-identical stem cell transplantation in patients with myeloid leukemias but not with acute lymphoblastic leukemia. Leukemia. 2007;21:2145-52.

26. Dunbar EM, Buzzeo MP, Levine JB, Schold JD, Meier-Kriesche HU, Reddy V. The relationship between circulating natural killer cells after reduced intensity conditioning hematopoietic stem cell transplantation and relapse-free survival and graft-versus-host disease. Haematologica. 2008;93(12):1852-8.

27. Cheung YK, Chappell R. Sequential designs for phase I clinical trials with late-onset toxicities. Biometrics, 56:1177-1182, 2000

28. Thall PF, Sung HG. Some extensions and applications of a Bayesian strategy for monitoring multiple outcomes in clinical trials. *Statistics in Medicine* 17:1563-80, 1998.

29. Kaplan, EL and Meier, P: Nonparametric estimation from incomplete observations. *J. American Statistical Association* 53:457-481, 1958.
