# Supplementary material for: Decrease post-transplant relapse using donor-derived expanded NK-cells
Source: Leukemia. Author manuscript; Available in PMC 2022 Oct 7. (PMC8727305; doi:10.1038/s41375-021-01349-4)

SUPPLEMENTAL INFORMATION

Table of Contents | Page

Table S1. Mass cytometry antibody panel- list of targets and heavy metal conjugants 2

Table S2. Recipient KIR characteristics 3

Table S3. Donor KIR characteristics 4

Table S4. Characteristics of the NK-cell products of 12 patients treated in the phase II extension study 5

Table S5. Causes of death in treatment and CIBMTR control group. 5

Table S7. Multivariable analysis of relapse, NRM, DFS, and OS: RIC controls vs. cases 7

Table S8. Multivariable analysis of relapse, NRM, DFS, and OS of MAC controls vs. cases 7

Table S9. Multivariable analysis of NRM, DFS, and OS: RIC controls vs. cases without DSA 8

Table S10. Multivariable analysis of NRM, DFS, and OS: MAC controls vs cases without DSA 8

Figure S1. FC21-NK cell expansion and product characteristics 9

Figure S2. Mass cytometry stochastic clustering by NK cell markers by individual patients and timepoints. 10

Table S1. Mass cytometry antibody panel- list of targets and heavy metal conjugants

151Eu_CD107a
153Eu_HLA_DR
154Sm_CD69
156Gd_CXCR3
165Ho_LAG3
175Lu_PERFORIN
142Ce_NKG2C
148Nd_CD161
152Sm_KIR2DL1
167Er_KIR3DL1
143Nd_CPARP
145Nd_CD137
149Sm_CD244
155Gd_CD27
158Gd_CXCR4
159Tb_NKP30
160Gd_CXCR6
164Dy_FAS
166Er_NKG2D
169Tm_NKG2A
170Er_TIM3
172Yb_NKP80
174Yb_CD94
161Dy_Ki67
146Nd_NKP44
157Gd_TIGIT
162Dy_NKP46
163Dy_CD56
171Yb_DNAM1
173Yb_KIR2DL2-DL3
176Yb_CD57
115In_CD45
113In_CD3
209Bi_CD16

Table S2. Recipient KIR characteristics

Patient	KIR2DS2	KIR2DL2	KIR2DL3	KIR2DL5A/B	KIR2DS3	KIR2DS5	KIR2DP1	KIR2DL1	KIR3DL1	KIR3DS1	KIR2DS4	KIR2DS1
1	-	-	+	+	-	+	+	+	+	+	+	+
3	-	-	+	-	-	-	+	+	+	-	+	-
4	+	+	+	-	-	-	+	+	+	-	+	-
5	-	-	+	-	-	-	+	+	+	-	+	-
6	+	+	+	+	+	+	+	+	+	+	+	+
7	+	+	+	+	+	-	+	+	+	+	+	+
8	-	-	+	-	-	-	+	+	+	-	+	-
9	-	-	+	-	-	-	+	+	+	-	+	-
10	-	-	+	+	+	-	+	+	+	-	+	-
11	-	-	+	-	-	-	+	+	+	-	+	-
12	-	-	+	-	-	-	+	+	+	-	+	-
13	-	+	+	+	+	-	+	+	+	-	+	-
14	+	+	+	+	+	+	+	+	+	-	+	-
15	+	+	+	+	-	+	+	+	+	+	+	+
16	+	+	+	+	+	-	+	+	+	-	+	-
17	-	-	+	-	-	-	+	+	+	-	+	-
18	-	-	+	-	-	-	+	+	+	-	+	-
19	+	+	+	+	+	+	+	+	+	+	+	+
20	+	+	+	-	-	-	+	+	+	-	+	-
21	+	+	+	+	+	-	+	+	+	+	+	-
22	+	+	+	+	-	+	+	+	+	-	+	+
23	+	+	+	+	+	-	+	+	+	+	+	+
24	-	-	+	-	-	-	+	+	+	-	+	-
25	+	+	+	+	+	-	+	+	+	-	+	-

Table S3. Donor KIR characteristics

Donor	KIR2DS2	KIR2DL2	KIR2DL3	KIR2DL5A/B	KIR2DS3	KIR2DS5	KIR2DP1	KIR2DL1	KIR3DL1	KIR3DS1	KIR2DS4	KIR2DS1
1	-	-	+	-	-	-	+	+	+	-	+	-
3	-	-	+	-	-	-	+	+	+	-	+	-
4	+	+	+	-	-	-	+	+	+	-	+	-
5	-	-	+	-	-	-	+	+	+	-	+	-
6	-	-	+	-	-	-	+	+	+	-	+	-
7	+	+	-	+	+	-	+	+	+	+	+	+
8	-	-	+	-	-	-	+	+	+	-	+	-
9	-	-	+	+	-	+	+	+	+	+	+	+
10	-	-	+	+	+	-	+	+	+	-	+	-
11	+	+	+	+	+	+	+	+	+	+	+	+
12	-	-	+	+	-	+	+	+	-	+	-	+
13	-	+	+	-	-	-	+	+	+	-	+	-
14	-	-	+	+	-	+	+	+	+	-	+	-
15	-	-	+	-	-	-	+	+	+	-	+	-
16	+	+	-	+	+	-	+	+	+	-	+	-
17	-	-	+	-	-	-	+	+	+	-	+	-
18	-	-	+	-	-	-	+	+	+	-	+	-
19	+	+	+	+	+	+	+	+	-	+	-	+
20	+	+	-	+	+	-	+	+	+	-	+	-
21	-	-	+	+	-	+	+	+	+	+	+	+
22	+	+	+	+	-	+	+	+	+	-	+	+
23	+	+	+	+	+	-	+	+	+	-	+	-
24	+	+	+	+	+	+	+	+	+	+	+	+
25	+	+	-	+	+	-	+	+	+	-	+	-

Table S4. Characteristics of the NK-cell products of 12 patients treated in the phase II extension study

Patient	Viability, %	Viable CD32+, APCs, %	CD3+ cells		CD3- CD(16,56+), NK cells, %	CD19+, B cells, %	CD14+ cells, monocytes, %
			%	x 10^5/Kg			
1	95	0.13	0.02	0.2	97.08	not detected	not detected
2	95	not detected	0.01	0.1	97.92	not detected	not detected
3	94	not detected	0.02	0.2	98.76	not detected	not detected
4	95	0.33	0.02	0.06	99.06	not detected	not detected
5	87	0.38	0.02	0.063	95.88	not detected	not detected
6	96	not detected	0.04	0.4	98.70	0.03	not detected
7	94	0.05	0.04	0.4	99.09	0.01	not detected
8	96	0.08	0.05	0.5	99.32	not detected	not detected
9	97	not detected	0.01	0.1	99.27	0.06	not detected
10	95	not detected	0.01	0.1	99.39	0.01	not detected
11	93	0.08	0.02	0.2	97.13	not detected	not detected
12	88	0.01	0.01	0.1	98.28	not detected	not detected

Table S5. Causes of death in treatment and CIBMTR control group.

	CASES (N=7)	CONTROLS (N=67)	MAC controls (N=31)	RIC controls (N=36)
Primary disease	0	36 (54%)	14 (45%)	22 (61%)
Graft failure	1 (14%) (secondary graft failure)	1 (1%)	1 (3%)	0
GVHD	1 (14%)	5 (7%)	3 (10%)	2 (6%)
Infection	2 (28%)	3 (4%)	1 (3%)	2 (6%)
Interstitial pneumonia/ARDS	1 (14%)	4 (6%)	3 (10%)	1 (3%)
Organ failure	0	9 (13%)	2 (6%)	7 (19%)
Second malignancy	0	1 (1%)	0	1 (3%)
Other causes	2 (28%) (1 unidentified cause of death, 1 hemorrhage)	8 (12%)	7 (23%)	1 (3%)

Table S6. Immunologic cell recoveries after transplant stratified by NK cell dose

	Total		Low dose* (N=5)		Intermediate dose* (N=7)		High dose* (N=12)		P value
	Mean	SD	Mean	SD	Mean	SD	Mean	SD	
**Day 30**									
WBC	4263	2399	4950	2397	3833	1969	4177	2836	0.773
Absolute lymphocytes	407	294	216	83	320	215	564	347	0.189
NK cells	636	964	122	136	284	305	1084	1282	0.064
CD3	91	145	89	22	40	35	136	207	0.397
CD4	47	81	23	1.4	24	29	74	115	0.848
CD8	41	77	66	24	15	11	56	113	0.217
CD19	2.8	2.7	1.5	0.7	3.1	3.2	2.9	2.7	0.909
CD25	12	13	6	7.1	11	8.3	15	17	0.824
CD45RO	67	130	14	7.8	23	21.1	119	183	0.876
CD45RA	2.7	3.1	0.5	0.7	3.8	3.4	NA	NA	0.140
**Day 90**									
WBC	3819	1694	3425	2410	4457	1786	3530	1830	0.421
Absolute lymphocytes	757	495	780	744	638	372	862	501	0.703
NK cells	269	240	190	132	256	164	314	324	0.695
CD3	442	616	1022	1109	209	164	365	443	0.689
CD4	170	220	215	314	111	32	196	269	0.737
CD8	264	485	781	870	100	148	161	301	0.335
CD19	82	102	97	114	116	115	50	88	0.325
CD25	19	26	15	4.2	42	38	6.3	5.1	0.005
CD45RO	155	190	58	41	111	59	228	258	0.424
CD45RA	13	20	2.3	1.7	25	24	NA	NA	0.148
**Day 180**									
WBC	5877	2767	6175	3309	5871	2219	5714	3346	0.988
Absolute lymphocytes	1649	1582	1926	804	1260	1341	1879	2164	0.231
NK cells	342	191	391	162	313	141	334	260	0.650
CD3	1254	1545	1082	440	1181	1296	1429	2271	0.577
CD4	401	297	326	50	378	241	470	435	0.881
CD8	794	1233	745	415	705	1042	900	1807	0.811
CD19	178	146	221	197	152	116	171	153	0.796
CD25	54	66	151	108	66	44	16	14	0.025
CD45RO	820	1327	415	213	630	411	1050	1815	0.716
CD45RA	39	20	30	18	57	NA	NA	NA	0.221
**Day 360**									
WBC	6392	2667	3500	NA	5566	2566	7700	2540	0.257
Absolute lymphocytes	2229	1142	1270	NA	1832	1097	2786	1087	0.149
NK	334	174	164	46	338	194	433	136	0.114
CD3	1193	609	769	254	1194	783	1147	503	0.285
CD4	608	319	365	149	557	229	806	381	0.126
CD8	514	357	370	152	559	475	556	353	0.572
CD19	496	341	383	171	609	389	450	393	0.673
CD25	35	19	24	NA	40	28	31	6	0.456
CD45RO	606	262	338	NA	526	177	802	295	0.143
CD45RA	NA	NA	NA	NA	NA	NA	NA	NA	NA

*Definitions: Low dose was <1 x10^7/Kg/dose; Intermediate dose was 1 x10^7 -3x10^7/Kg/dose; High dose was 1 x10^8/Kg/dose

Abbreviations: SD: standard deviation, NA: not available

Table S7. Multivariable analysis of relapse, NRM, DFS, and OS: RIC controls vs. cases

	Number Events / Evaluable	Hazard Ratio (95% Confidence Interval)	P-value
**DFS**			
Cases	8/24	1.00[a]	
RIC Controls	48/79	2.28 (1.08 - 4.82)	0.03
**NRM**			
Cases	7/24	1.00[a]	
RIC Controls	12/79	0.60 (0.22 - 1.68)	0.33
**Relapse**			
Cases	1/24	1.00[a]	
RIC Controls	36/79	14.18 (1.75 - 115.00)	0.013
**OS**			
Cases	7/24	1.00[a]	
RIC Controls	36/79	1.66 (0.71 - 3.88)	0.24

[a] Reference group

Variables considered for analysis: recipient age, recipient gender, recipient race and ethnicity, HCT-CI, performance score, CMV serostatus, graft type, year of transplant

There were no significant factors in the DFS, NRM, relapse, and OS models. The results shown are from the models with only the main effect.

Table S8. Multivariable analysis of relapse, NRM, DFS, and OS of MAC controls vs. cases

	Number Events / Evaluable	Hazard Ratio (95% Confidence Interval)	P-value
**DFS**			
Cases	8/24	1.00[a]	
MAC Controls	39/81	1.55 (0.67 - 3.55)	0.30
**NRM**			
Cases	7/24	1.00[a]	
MAC Controls	16/81	0.73 (0.29 - 1.85)	0.51
**Relapse**			
Cases	1/24	1.00[a]	
MAC Controls	23/81	7.24 (0.95 - 55.16)	0.06
**OS**			
Cases	7/24	1.00[a]	
MAC Controls	31/81	1.31 (0.56 - 3.04)	0.39

[a] Reference group

Variables considered for analysis: recipient age, recipient gender, recipient race and ethnicity, HCT-CI, performance score, cmv serostatus, graft type, year of transplant

There were no significant factors in the DFS, NRM, relapse, and OS models. The results shown are from the models with only the main effect.

Table S9. Multivariable analysis of NRM, DFS, and OS: RIC controls vs. cases without DSA

	**Number Events / Evaluable**	**Hazard Ratio (95% Confidence Interval)**	**P-value**
**DFS**			
Cases	5/19	1.00[a]	
RIC Controls	36/62	3.33 (1.30 - 8.52)	0.012
**NRM**			
Cases	5/19	1.00[a]	
RIC Controls	8/62	0.63 (0.21 - 1.95)	0.43
**OS**			
Cases	5/19	1.00[a]	
RIC Controls	28/62	1.84 (0.67 - 5.04)	0.24

[a] Reference group

Variables considered for analysis: recipient age, recipient gender, recipient race and ethnicity, HCT-CI, performance score, cmv serostatus, graft type, year of transplant

There were no significant factors in the DFS, NRM, relapse, and OS models. The results shown are from the models with only the main effect.

Table S10. Multivariable analysis of NRM, DFS, and OS: MAC controls vs cases without DSA

	**Number Events / Evaluable**	**Hazard Ratio (95% Confidence Interval)**	**P-value**
**DFS**			
Cases	5/19	1.00[a]	
MAC Controls	30/63	2.08 (0.87 - 4.95)	0.10
**NRM**			
Cases	5/19	1.00[a]	
MAC Controls	13/63	0.90 (0.35 - 2.29)	0.83
**OS**			
Cases	5/19	1.00[a]	
MAC Controls	23/63	1.44 (0.61 - 3.43)	0.41

[a] Reference group

Variables considered for analysis: recipient age, recipient gender, recipient race and ethnicity, HCT-CI, performance score, cmv serostatus, graft type, year of transplant

There were no significant factors in the DFS, NRM, relapse, and OS models. The results shown are from the models with only the main effect.

Figure S1. FC21-NK cell expansion and product characteristics

Panel (A) shows fold expansion was calculated as the ratio of the NK cell content (CD3-CD56+) of the final product for infusion to the NK cell content of the starting MNC product after CD3 depletion and adjusted for occurrences in which only a portion of the product was processed, cultured, or carried forward in culture.

Panel (B) shows viability and NK cell content (CD3-CD56+) of the final fresh product prepared for infusion.

Panel (C) shows residual FC21 (CD19 or CD32+, and CD56-), FC21 or B cell (CD19+), and T cell (CD3+) content in the final NK cell product.

Panel (D) shows viability and recovery of 31 cryopreserved NK cell products assessed after thawing and preparing for infusion. Bars and whiskers represent median ± interquartile range.

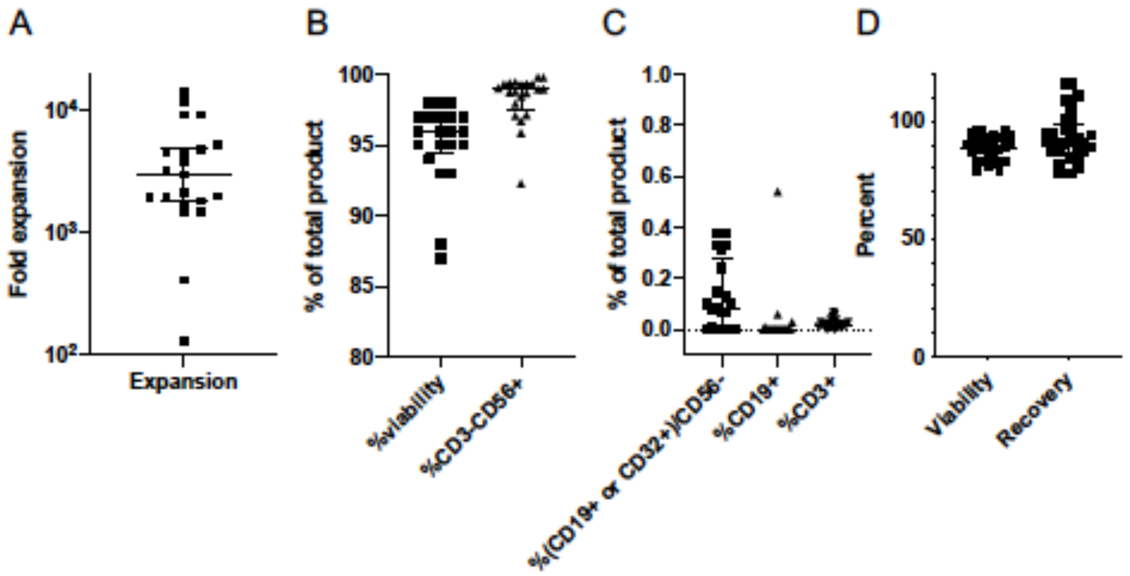

Figure S2. Mass cytometry stochastic clustering by NK cell markers by individual patients and timepoints.

Peripheral blood was obtained at the indicated timepoints, and mononuclear cells (MNC) were isolated, processed, and assessed for 34 parameters (Supplemental Table S1) along with healthy subject MNC and expanded FC21-NK cell products as reference samples. Samples were gated on live cells, singlets, and cPARP-/CD45+ events. ViSNE plots were constructed by clustering on eight parameters. Shown are plots from all patients at all timepoints, healthy subjects, and NK cell products, showing expression of CD56 as a reference for each sample. Cluster 1 (bottom left) consists of $CD3^{+}$ T cells, Cluster 2 (top middle) of $CD3^{-}$ $CD56^{dim}NKG2D^{dim}CD57^{+}$ "standard" NK cells, Cluster 3 (top right) of $CD56^{br}NKG2D^{br}NKp46^{br}CD57^{-}$ NK cells corresponding to the phenotype of the infused FC21-NK cell product, and Cluster 4 (bottom middle) of all remaining cells. Cluster 3 identifies a unique phenotypic signature associated with the FC21-NK cells that is not present in healthy subjects and persists in patients after adoptive transfer.

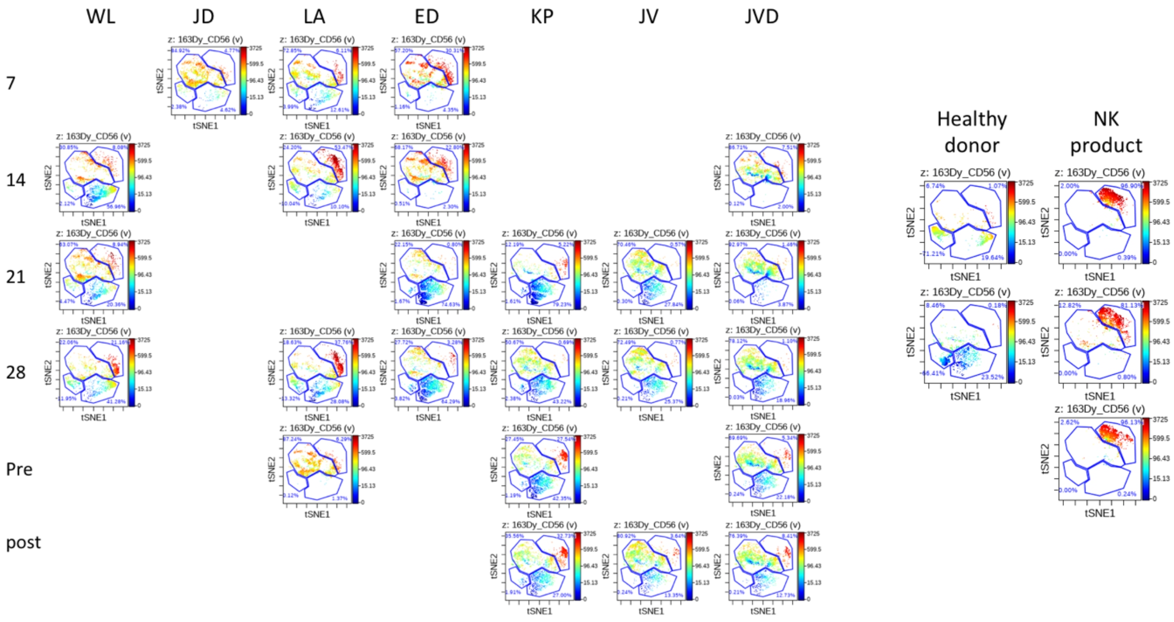

Supplement: 1739240_Sup_Info [file NIHMS1739240-supplement-1739240_Sup_Info.pdf]
